# Overview of the ID, EPI and REL tasks of BioNLP Shared Task 2011

**DOI:** 10.1186/1471-2105-13-S11-S2

**Published:** 2012-06-26

**Authors:** Sampo Pyysalo, Tomoko Ohta, Rafal Rak, Dan Sullivan, Chunhong Mao, Chunxia Wang, Bruno Sobral, Jun'ichi Tsujii, Sophia Ananiadou

**Affiliations:** 1 School of Computer Science, University of Manchester, Manchester, UK; 2 National Centre for Text Mining, University of Manchester, Manchester, UK; 3 Department of Computer Science, University of Tokyo, Tokyo, Japan; 4 Virginia Bioinformatics Institute, Virginia Tech, Blacksburg, Virginia, USA; 5 Microsoft Research Asia, Beijing, China

## Abstract

We present the preparation, resources, results and analysis of three tasks of the BioNLP Shared Task 2011: the main tasks on Infectious Diseases (ID) and Epigenetics and Post-translational Modifications (EPI), and the supporting task on Entity Relations (REL). The two main tasks represent extensions of the event extraction model introduced in the BioNLP Shared Task 2009 (ST'09) to two new areas of biomedical scientific literature, each motivated by the needs of specific biocuration tasks. The ID task concerns the molecular mechanisms of infection, virulence and resistance, focusing in particular on the functions of a class of signaling systems that are ubiquitous in bacteria. The EPI task is dedicated to the extraction of statements regarding chemical modifications of DNA and proteins, with particular emphasis on changes relating to the epigenetic control of gene expression. By contrast to these two application-oriented main tasks, the REL task seeks to support extraction in general by separating challenges relating to part-of relations into a subproblem that can be addressed by independent systems. Seven groups participated in each of the two main tasks and four groups in the supporting task. The participating systems indicated advances in the capability of event extraction methods and demonstrated generalization in many aspects: from abstracts to full texts, from previously considered subdomains to new ones, and from the ST'09 extraction targets to other entities and events. The highest performance achieved in the supporting task REL, 58% F-score, is broadly comparable with levels reported for other relation extraction tasks. For the ID task, the highest-performing system achieved 56% F-score, comparable to the state-of-the-art performance at the established ST'09 task. In the EPI task, the best result was 53% F-score for the full set of extraction targets and 69% F-score for a reduced set of core extraction targets, approaching a level of performance sufficient for user-facing applications. In this study, we extend on previously reported results and perform further analyses of the outputs of the participating systems. We place specific emphasis on aspects of system performance relating to real-world applicability, considering alternate evaluation metrics and performing additional manual analysis of system outputs. We further demonstrate that the strengths of extraction systems can be combined to improve on the performance achieved by any system in isolation. The manually annotated corpora, supporting resources, and evaluation tools for all tasks are available from http://www.bionlp-st.org and the tasks continue as open challenges for all interested parties.

## Background

The biomedical scientific literature is growing at an exponential rate, far outstripping the capacity of individual researchers to fully process in any but the narrowest of subfields. To address the challenges of information overload and to improve access to the wealth of knowledge in this literature, there have been substantial efforts over the previous 15 years to develop automatic methods for the analysis of medical and biomolecular scientific publications [1,2].

Much of this work has focused on information extraction (IE) and text mining, applying natural language processing (NLP) methods to analyse domain texts, extract structured, computer-readable representations of key information, and compile extracted information into knowledge bases. Until recently, domain IE efforts concentrated primarily on foundational tasks, such as entity mention detection, and on the extraction of simple representations of entity associations, most typically the detection of mentions of names of proteins and the extraction of protein mention pairs representing protein-protein interactions.

However, in recent years there has been increasing interest in the application of more expressive representations in domain IE to address the requirements of tasks such as pathway curation, Gene Ontology term annotation, and semantic literature search [3,4]. To support the development and evaluation of methods for such tasks, a number of recently introduced corpus resources have been manually annotated using *event representations *that capture structured associations of arbitrary numbers of participants in specific roles [5-10]. The community took a decisive step toward the introduction of practical tools capable of extracting information using such representations in the BioNLP Shared Task 2009 (BioNLP ST'09) [11,51].

Shared tasks have been instrumental in the development of general domain IE technology by introducing new tasks, resources and evaluation standards [12,13]. Also in the biomedical domain, shared tasks such as JNLPBA [14], LLL [15], TREC Genomics [16] and BioCreative [17-19] have played a central role in focusing the efforts of the community to new timely tasks and challenges. The BioNLP ST'09, the first shared task in its series, sought to advance the state of the art in structured event extraction by providing a shared task definition and annotated data as well as evaluation criteria and tools for the task. The task met with enthusiastic response from the community: 24 groups participated in the task, proposing a variety of approaches for automatic event extraction. Interest in event extraction continued past the original shared task, whose data and setup have supported further advances in extraction methods and the introduction of automatically annotated literature-scale resources [20-26].

Although successful in introducing structured event representations to the general community and promoting the development of practically applicable methods for event extraction, the resources of the BioNLP ST'09 were somewhat limited in their scope. The task data was prepared on the basis of the GENIA corpus [6,27], an annotated resource of publication abstracts in the domain of *transcription factors in human blood cells*, and the event types targeted in the task were chosen by relevance to its topics. These limitations raised the question whether the findings of the shared task and the methods introduced to address the task can generalize beyond this narrow domain. To address such questions, *generalization *was chosen as the main theme of the second event in the series, the BioNLP Shared Task 2011 (BioNLP ST'11) [28]. This was emphasized in task selection and design and data preparation, targeting new domains, an extended set of extraction targets, and new text types, including full-text articles.

In this paper, we present three of the eight tasks in BioNLP ST'11, including two tasks that attracted the widest participation among newly introduced main tasks, EPI and ID. The Epigenetics and Post-translational Modifications (EPI) task focuses on events relating to epigenetic change and encompasses also common protein post-translational modifications, reactions that are critical for the control or gene expression and protein function. The Infectious Diseases (ID) task concerns the biomolecular mechanisms of infection, virulence and resistance, focusing in particular on the functions of a class of signaling systems that are ubiquitous in bacteria but as of yet incompletely understood. In addition to these two main tasks, we introduce the Entity Relations (REL) supporting task, which seeks to assist extraction in general by separating challenges relating to part-of relations into a subproblem that can be addressed by independent systems whose analyses can then be used to support the recognition of various event extraction targets.

Each of these tasks follows the general design of the BioNLP ST'09, providing participants with an extensive, fully annotated corpus with manually curated examples of the extraction targets for method development and training, with evaluation of final submissions received from participants against a separate held-out test set prepared in similar fashion.

We extend on the EPI, ID and REL task results previously reported in the BioNLP Shared Task 2011 workshop proceedings [29-32] in particular in performing further analyses of the outputs of the participating systems, placing specific emphasis on aspects of event extraction system performance relating to real-world applicability, considering alternate evaluation metrics and performing additional manual analysis of system outputs. We further demonstrate that the strengths of extraction systems can be combined to improve on the performance achieved by any system in isolation.

In the following, we first briefly motivate each of the EPI, ID and REL tasks and introduce the task setting. We then describe the task data annotation and evaluation criteria before presenting the results of each task. Finally, we present an extended analysis of the outputs of the participating systems.

### EPI task

The Epigenetics and Post-translational Modifications (EPI) task is an information extraction task focusing on events relating to epigenetic change, including DNA methylation and histone methylation and acetylation (see e.g. [33,34]), as well as other common protein post-translational modifications (PTMs) [35]. PTMs are chemical modifications of the amino acid residues of proteins, and DNA methylation a parallel modification of the nucleotides on DNA. While these modifications are chemically simple reactions and can thus be straightforwardly represented in full detail, they have a crucial role in the regulation of gene expression and protein function: the modifications can alter the conformation of DNA or proteins and thus control their ability to associate with other molecules, making PTMs key steps in protein biosynthesis for introducing the full range of protein functions. For instance, protein phosphorylation - the attachment of phosphate - is a common mechanism for activating or inactivating enzymes by altering the conformation of protein active sites [36,37], and protein ubiquitination - the post-translational attachment of the small protein ubiquitin - is the first step of a major mechanism for the destruction (breakdown) of many proteins [38].

Many of the PTMs targeted in the EPI task involve modification of histone, a core protein that forms an octameric complex that has a crucial role in packaging chromosomal DNA. The level of methylation and acetylation of histones controls the tightness of the chromatin structure, and only "unwound" chromatin exposes the gene packed around the histone core to the transcriptional machinery. Since histone modification is of substantial current interest in epigenetics, we designed aspects of the EPI task to capture the full detail in which histone modification events are stated in text. Finally, the DNA methylation of gene regulatory elements controls the expression of the gene by altering the affinity with which DNA-binding proteins (including transcription factors) bind, and highly methylated genes are not transcribed at all [39,40]. DNA methylation can thus "switch off" genes in a way that is reversible through DNA demethylation.

**Figure 1:**
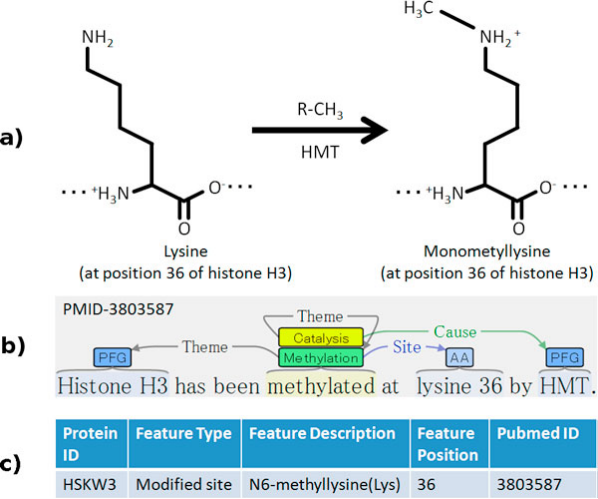
**Three views of protein methylation**. a) Chemical formula b) Event representation c) Modification database entry.

The specificity with which protein modifications can be described in text makes them promising IE targets (see Figure 1), and there have been many studies of automatic extraction of PTMs from the scientific literature in support of modification database curation [41-44]. However, these have generally targeted only single PTM types such as phosphorylation or ubiquitination, frequently using highly customized rule-based systems that are not readily adaptable to other extraction targets. The BioNLP ST'09 involved the extraction of nine event types including one PTM type, PHOSPHORYLATION, which was found to be the single most reliably extracted event type in the task, with the best-performing system for the type achieving 83% F-score in its extraction [45]. The results suggest both that the event representation is well applicable to PTM extraction and that current extraction methods are capable of reliable PTM extraction. Many of the most successful systems participating in the ST'09 further involved general machine learning-based approaches, suggesting that their scope could be extended to PTM extraction more broadly. The EPI task follows up on these opportunities, introducing specific, strongly biologically motivated extraction targets that are expected to be both feasible for high-accuracy event extraction, relevant to the needs of present-day molecular biology, and closely applicable to biomolecular database curation needs.

### ID task

The Infectious Diseases (ID) task is an event extraction task focusing on the biomolecular mechanisms of infectious diseases. The task concentrates on the specific domain of two-component systems (TCSs, or two-component regulatory systems), a mechanism widely used by bacteria to sense and respond to the environment [46]. Typical TCSs consist of two proteins, a membrane-associated sensor kinase and a cytoplasmic response regulator. The sensor kinase monitors changes in the environment while the response regulator mediates an adaptive response, usually through differential expression of target genes [47]. TCSs have many functions, but those of particular interest for infectious disease researchers include virulence, response to antibiotics, quorum sensing, and bacterial cell attachment [48]. Not all TCS functions are well known: in some cases, TCSs are involved in metabolic processes that are difficult to precisely characterize [49]. TCSs are of interest also as drugs designed to disrupt TCSs may reduce the virulence of bacteria without killing it, thus avoiding the potential selective pressure of antibiotics lethal to some pathogenic bacteria [50]. Information extraction techniques may support better understanding of these fundamental systems by identifying and structuring the molecular processes underlying two component signaling.

The ID task seeks to support efforts to build a systemic understanding of the molecular-level pathways relating to these mechanisms of infectious diseases by adapting the BioNLP ST'09 event extraction model to domain scientific publications. The adaptation of the model originally introduced to represent biomolecular events relating to transcription factors in human blood cells to a domain that centrally concerns both bacteria and their hosts involves a variety of novel challenges, such as events concerning whole organisms, the chemical environment of bacteria, prokaryote-specific concepts (e.g. regulons as units of gene regulation), as well as the effects of biomolecules on larger-scale processes involving hosts, such as virulence. In addition to supporting an application of significant public health interest, the ID task also provides opportunities to study the ability of event extraction technology to generalize in a number of aspects.

### REL supporting task

The Entity Relations (REL) supporting task focuses on the extraction of specific binary relations between biomolecular entities. The motivation for the task draws in part from analysis of the results of the BioNLP ST'09, which suggested that events that involve coreference or entity relations represent particular challenges for extraction [51]. To help address these challenges and encourage modular extraction approaches, increased sharing of successful solutions, and an efficient division of labor, the two were separated into independent supporting tasks on Coreference (CO) [52,53] and Entity Relations [32] for BioNLP ST'11. To allow participants in main tasks to benefit from successful approaches to the supporting tasks, the ST'11 was arranged in two distinct stages, with supporting tasks carried out before the main tasks.

## Methods

In this section, we introduce the general representation of the IE tasks, the specific realization of this representation applied in each task, and the task evaluation criteria.

### Representation

While the EPI, ID and REL tasks differ substantially in the specifics of their extraction targets, the three share the same basic representation of extracted information, an extension of the representation introduced for the BioNLP ST'09 [11] and applied also in the ST'11 GE task [53,54]. In this section, we first present the shared aspects of this event representation, in particular the four annotation primitives *entities*, *relations*, *events *and *event modifications*, briefly illustrate the format in which these are stored in the task, and then describe the specific set of annotations involved in each of the tasks.

#### Entities

**Figure 2:**
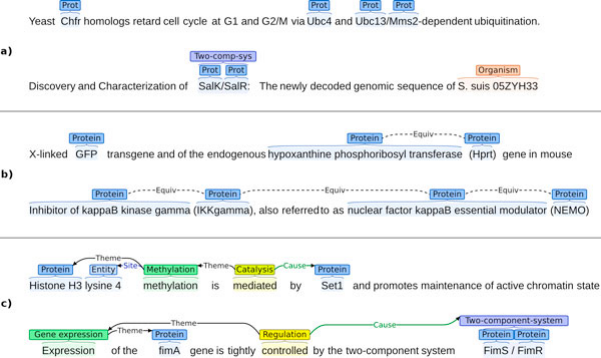
**Illustration of annotations**. a) Entity annotation b) Equiv annotation c) Event annotation. PROT abbreviates for PROTEIN and TWO-COMP-SYS for TWO-COMPONENT SYSTEM. (Annotation visualizations generated using BRAT[106,107]).

All of the tasks build on the basis of entity annotations, which capture mentions of entities of interest in text using a simple typed-span representation. Each entity annotation consists of a type (e.g. PROTEIN) and a *(start*, *end) *offset pair identifying the span of text containing the entity mention. The entity annotations thus mark contiguous sequences of characters. All entity annotations further follow the constraint that no two entities of the same type overlap in their spans, and that the spans of no two overlapping annotations cross. By contrast, entity annotation spans may be nested so that one span completely contains another. Figure 2a shows examples of entity annotation.

Entity mention detection and normalization are arguably the most frequently studied IE-related tasks in the domain, and the target of numerous previous and ongoing shared tasks [14,55-58]. Further, a wealth of systems addressing these tasks have been introduced (e.g. [59-62]). To focus on the novel aspects of event extraction, the BioNLP Shared Task series has adopted the general policy of providing task participants with manual "gold standard" annotation identifying the primary entities relevant to each task as a starting point for extraction, thus isolating effects of entity mention detection from IE performance.

The three tasks presented in this paper share the definitions of two entity types, PROTEIN and ENTITY, defined similarly as for the BioNLP ST 2009. Mentions of specific names of genes and gene products are annotated as PROTEIN in all tasks, with some task-specific exceptions to the precise scope of the annotation (see the sections on each task). Gold annotation for PROTEIN entities is provided to participants in all tasks. The generic type ENTITY, by contrast, is defined for marking additional entity annotations generated by participants, such as the specific protein domains or DNA regions involved in modification or binding events. Annotations of this type are only provided in training data, and must thus be detected for test data by systems addressing the full main tasks or the supporting task. The non-specific type ENTITY is selected in part to reduce the demands of this entity recognition component of the tasks by removing the need to differentiate between specific types.

#### Relations

Relations are typed binary associations of entities and may be either directed or undirected. While relations are only a target of extraction in the REL task, all of the three tasks involve a specific relation, EQUIV. This is a binary, symmetric, transitive relation that defines two entities to be equivalent [63]. The relation is used in the gold annotation to mark local aliases such as the full and abbreviated forms of a protein name as referring to the same real-world entity. In evaluation, references to any of a set of equivalent entities are treated identically. Figure 2b shows examples of equiv annotation.

In addition to the general EQUIV relation, the REL task defines directed part-of relations that are the targets of extraction in the supporting task, introduced in the section defining the task.

#### Events

Events are typed, *n*-ary associations of entities or other events, each identified as participating in a specific role. Events are bound to specific expressions in text (the *event trigger *or *text binding*) and are primary objects of annotation; that is, annotations may refer to event annotations. Event triggers identify the word or words stating the occurrence of the event in text. Like entities, triggers are represented using a (*start*, *end*) offset pair. Event types (e.g. BINDING, ACETYLATION) are drawn from a fixed set separately defined for each task. Each event typically takes one or more arguments (participants in specific roles): for example, an ACETYLATION event may be defined as requiring a single *Theme *identifying the PROTEIN entity that is acetylated. Events may involve other events as participants, thus creating complex event structures. For example, a REGULATION event may have a BINDING event as its *Theme*, thus specifying a compound "regulation of binding" event. Figure 2c shows examples of event annotation.

The main tasks differ in the details of event arguments, but share the definition of the basic core argument roles *Theme *and *Cause *as well as the additional argument role *Site*. As the terms suggest, *Theme *identifies the participant or participants that undergo the primary effects of the event, and *Cause *a participant that causes the event to occur. *Site *identifies a specific part of another participant that is involved in the event, such as the modified residue in a PHOSPHORYLATION event. Event arguments may be specified as being either mandatory or optional, where mandatory arguments must be identified for an event to be extracted. Events typically take a mandatory *Theme*, reflecting a specificity constraint on the extracted information: while statements regarding e.g. the phosphorylation of specific proteins are targeted for extraction, statements regarding phosphorylation in general are not.

The event arguments vary by event type and task, and the specification of event types and arguments largely defines the differences between the different main tasks of the BioNLP ST'11.

#### Event modifications

Event modification annotations are used to specify further aspects of event statements beyond the core propositional content, for example identifying an event as being negated. Event modifications are represented as simple binary "flags" attached to events. Both of the main event extraction tasks EPI and ID follow the BioNLP ST'09 setting in defining two event modification extraction targets: NEGATION and SPECULATION. The former marks an event as being explicitly negated (e.g. *H2A is not methylated*) and the latter as stated in a speculative context (e.g. *H2A may be methylated*). An event may be simultaneously marked as both negated and speculated (e.g. *H2A may not be methylated*). Unlike in the representation used for events and the cue-scope model applied for negation and speculation annotation in e.g. the BioScope corpus and the CoNLL 2010 shared task [64,65], no "trigger expressions" are marked for event modifications.

#### Format

**Figure 3:**
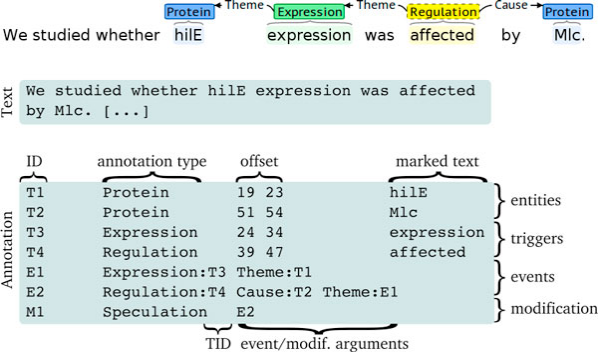
**Illustration of file format for a simple event structure**. "TID" abbreviates for "trigger ID".

The above presentation of the represented information content abstracts away the specific file format in which this information is stored in the task. While secondary to this information content, the specifics of the format may be of interest for assessing the technical requirements of the task; we thus include in Figure 3 an illustration of the applied file format. In brief, this is a standoff annotation format in which all references to the text are stored as offsets. Each annotation is given an ID that is used to refer to that annotation, and the ID assignment follows a simple scheme to assist identifying entity types (e.g. IDs beginning with "E" for events). For a detailed description of the format, we refer to the BioNLP ST'09 overview [51].

### EPI task setting

The EPI task focuses on the extraction of information on statements regarding change in the chemical modification state of proteins and DNA. The task involves the two generally applied entity types PROTEIN and ENTITY, where annotations of the PROTEIN type are provided as part of the input. By contrast to its standard entity definition, the EPI task introduces considerable novelty in the targeted events, involving a total of 14 event types and two participant roles not considered in other BioNLP ST'11 tasks. Table 1 summarizes the targeted event types and their arguments. In addition to the standard *Theme*, *Cause *and *Site*, EPI defines the task-specific arguments *Sidechain *and *Contextgene*.

**Table 1:** EPI event types and their arguments

Type	Core arguments	Additional arguments
HYDROXYLATION	*Theme*(PROTEIN)	*Site*(ENTITY)
DEHYDROXYLATION	*Theme*(PROTEIN)	*Site*(ENTITY)
PHOSPHORYLATION	*Theme*(PROTEIN)	*Site*(ENTITY)
DEPHOSPHORYLATION	*Theme*(PROTEIN)	*Site*(ENTITY)
UBIQUITINATION	*Theme*(PROTEIN)	*Site*(ENTITY)
DEUBIQUITINATION	*Theme*(PROTEIN)	*Site*(ENTITY)
DNA METHYLATION	*Theme*(PROTEIN)	*Site*(ENTITY)
DNA DEMETHYLATION	*Theme*(PROTEIN)	*Site*(ENTITY)
GLYCOSYLATION	*Theme*(PROTEIN)	*Site*(ENTITY), *Sidechain*(ENTITY)
DEGLYCOSYLATION	*Theme*(PROTEIN)	*Site*(ENTITY), *Sidechain*(ENTITY)
ACETYLATION	*Theme*(PROTEIN)	*Site*(ENTITY), *Contextgene*(PROTEIN)
DEACETYLATION	*Theme*(PROTEIN)	*Site*(ENTITY), *Contextgene*(PROTEIN)
METHYLATION	*Theme*(PROTEIN)	*Site*(ENTITY), *Contextgene*(PROTEIN)
DEMETHYLATION	*Theme*(PROTEIN)	*Site*(ENTITY), *Contextgene*(PROTEIN)

CATALYSIS	*Theme*(Event), *Cause*(PROTEIN)	

The type of entity allowed as argument is specified in parenthesis.

**Figure 4:**
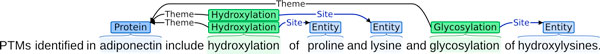
**Example EPI event annotation**. Three stated post-translational modifications of adiponectin are captured through three events, two of which differ only in their *Site *arguments. (Simplied from PMID 16497731).

*Sidechain*, specific to GLYCOSYLATION and DEGLYCOSYLATION among the targeted events, identifies the moiety attached or removed in the event (in glycosylation, the sugar). Note that while arguments similar to *Sidechain *could be defined for other event types also, their extraction would provide no additional information: the attached molecule is always acetyl in acetylation, methyl in methylation, etc. *Contextgene*, specific to ACETYLATION and METHYLATION events and their reverse reactions, identifies the gene whose expression is controlled by these modifications. This argument applies specifically for histone protein modification: the modification of the histones that form the nucleosomes that structure DNA are key to the epigenetic control of the expression of the genes encoded in that segment of DNA. *Theme *is required for all events in the EPI task, but the *Site*, *Sidechain *and *Contextgene *arguments are not mandatory, and should only be extracted when explicitly stated. For CATALYSIS events, representing the catalysis of protein or DNA modification by another protein, both *Theme *and Cause are mandatory. Figure 4 illustrates some of the EPI task extraction targets.

While the EPI task setting has few extraction targets in common with the BioNLP ST'09 and the ST'11 GE task, its entity types follow the same scheme as these tasks and the general definition aims to preserve compatibility with their setting. The basic modification events in EPI are defined similarly to the PHOSPHORYLATION event type targeted in ST'09, and while CATALYSIS is a new event type, it is related to the ST'09 POSITIVE REGULATION type by a class-subclass relation: any CATALYSIS event is a POSITIVE REGULATION event in the ST'09 task terms (but not vice versa).

As described in the section on representation, the EPI task targets also the two event modifications NEGATION and SPECULATION, and involves EQUIV relations in its evaluation. The task extraction targets do not include any relations.

### ID task setting

The ID task concerns the molecular mechanisms of infectious diseases, which involve various associations between multiple types of molecular entities, disease-causing microorganisms and other organisms undergoing the diseases. To support the extraction of information from domain publications, the task extends the basic entity types with multiple new categories. The ID event types extend on those defined in the BioNLP ST'09, broadening the scope of previously defined entities to encompass the new entity types and introducing a new class of events, high-level biological processes.

In addition to PROTEIN, the ID task defines four additional types of *core entities*: TWO-COMPONENT-SYSTEM, REGULON-OPERON, CHEMICAL and ORGANISM. As in the other tasks, mentions of names of genes and their products (RNA and proteins) are annotated with the PROTEIN type. Two-component systems, consisting of two proteins, frequently have names derived from the names of the proteins involved (e.g. *PhoP*-*PhoR *or *SsrA*/*SsrB*). Mentions of TCSs are annotated as TWO-COMPONENT-SYSTEM, nesting PROTEIN annotations if present. Regulons and operons are collections of genes whose expression is jointly regulated. Like the names of TCSs, their names may derive from the names of the involved genes and proteins, and are annotated as embedding PROTEIN annotations when they do. The annotation does not differentiate between the two, marking both with a single type REGULON-OPERON.

In addition to these three classes relating to genes and proteins, the core entity annotation recognizes the classes CHEMICAL and ORGANISM. All mentions of formal and informal names of atoms, inorganic compounds, carbohydrates and lipids as well as organic compounds other than amino acid and nucleic acid compounds (i.e. gene/protein-related compounds) are annotated as CHEMICAL. Mentions of names of families, genera, species and strains, as well as non-name references with comparable specificity are annotated as ORGANISM. The recognition of these core entities is not part of the ID task: gold annotation for these entities is provided to participants also for test data. As in the other tasks, the non-specific type ENTITY is defined for marking entities that specify additional details of events, ENTITY annotations are not provided for test data and must be detected by participants addressing the full task.

The primary extraction targets of the ID task are the event types summarized in Table 2. These are a superset of those targeted in the BioNLP ST'09 and the ST'11 GE task [54]. This design makes it possible to study aspects of domain adaptation by having the same extraction targets in two subdomains of biomedicine, that of transcription factors in human blood cells (GE) and infectious diseases. The events in the ID task extend on those of GE in the inclusion of additional entity types as participants in previously considered event types and the introduction of the new PROCESS type. The semantics of these events are defined (as in GE) with reference to the community-standard Gene Ontology [66]. We refer to [6,11] for the ST'09/GE definitions.

**Table 2:** ID event types and their arguments

Type	Core arguments	Additional arguments
GENE EXPRESSION	*Theme*(PROTEIN or REGULON-OPERON)	
TRANSCRIPTION	*Theme*(PROTEIN or REGULON-OPERON)	
PROTEIN CATABOLISM	*Theme*(PROTEIN)	
PHOSPHORYLATION	*Theme*(PROTEIN)	*Site*(ENTITY)
LOCALIZATION	*Theme*(Core entity)	*AtLoc*(ENTITY), *ToLoc*(ENTITY)
BINDING	*Theme*(Core entity)+	*Site*(ENTITY)+
PROCESS	*Participant*(Core entity)?	
REGULATION	*Theme*(Core entity/Event), *Cause*(Core entity/Event)?	*Site*(ENTITY), *CSite*(ENTITY)
POSITIVE REGULATION	*Theme*(Core entity/Event), *Cause*(Core entity/Event)?	*Site*(ENTITY), *CSite*(ENTITY)
NEGATIVE REGULATION	*Theme*(Core entity/Event), *Cause*(Core entity/Event)?	*Site*(ENTITY), *CSite*(ENTITY)

The type of entity allowed as argument is specified in parenthesis. "Core entity" is any of PROTEIN, TWO-COMPONENT-SYSTEM, REGULON-OPERON, CHEMICAL, or ORGANISM. Arguments that can be filled multiple times marked with "+", non-mandatory core arguments with "?" (all additional arguments are non-mandatory).

The definitions of the first four types in Table 2 are otherwise unchanged from the ST'09 definitions except that GENE EXPRESSION and TRANSCRIPTION extend on the former definition in recognizing REGULON-OPERON as an alternative unit of expression. LOCALIZATION, taking only PROTEIN type arguments in the ST'09 definition, is allowed to take any core entity argument. This expanded definition remains consistent with the scope of the corresponding GO term (GO:0051179). BINDING is similarly extended, giving it a scope largely consistent with GO:0005488 (binding) but also encompassing GO:0007155 (cell adhesion) (e.g. a bacterium binding another) and protein-organism binding. The three regulation types (REGULATION, POSITIVE REGULATION, and NEGATIVE REGULATION) likewise allow the new core entity types as arguments, but their definitions are otherwise unchanged from those in ST'09, that is, the GENIA ontology definitions. As in these resources, regulation types are used not only for the biological sense but also to capture statements of general causality [6]. As in ST'09, all events of types discussed above require a *Theme *argument: only events involving an explicitly stated theme (of an appropriate type) should be extracted. All other arguments are optional.

**Figure 5:**
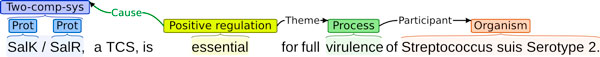
**Example ID event annotation**. The association of a TCS with an organism is captured through an event structure involving a PROCESS ("virulence") and POSITIVE REGULATION. Regulation types are used to capture also statements of general causality such as "is essential for" here. (Simplified from PMC ID 2358977).

The PROCESS type, new to ID, is used to annotate high-level processes such as virulence, infection and resistance that involve infectious organisms. This type differs from the others in that it has no mandatory arguments: the targeted processes should be extracted even if they have no explicitly stated participants, reflecting that they are of interest even without the further specification. When stated, the involved participants are captured using the generic role type *Participant*. Figure 5 shows an illustration of some of the ID task extraction targets.

We term the first five event types in Table 2 taking exactly one *Theme *argument as their core argument *simple events*. In analysis we further differentiate *non*-*regulation events *(the first seven) and *regulation *(the last three). The latter category is known to represent particular challenges for extraction in involving nested event structures where events take events as arguments.

The ID task event modifications and relations are defined similarly as for GE and EPI: the two modifications NEGATION and SPECULATION are targeted for extraction in the full task, and EQUIV relations are used in evaluation, but no relations are included among extraction targets.

#### REL task setting

The REL task aims to support the main event extraction tasks by isolating the recognition of entity relations as an independent subtask whose results can be used to resolve event structures. Following the results and analysis from previous studies [67,68], in the design of the REL task we chose to limit the task specifically to relations involving a gene/protein named entity (NE) and one other entity. Fixing one entity involved in each relation to an NE helps assure that the relations are "anchored" to real-world entities, and the specific choice of the gene/protein NE class further provides a category with several existing systems and substantial ongoing efforts addressing the identification of those referents through named entity recognition and normalization.

The recognition of biologically relevant associations of gene/protein NEs is a key focus of the main event extraction tasks of the shared task. As marking identifying mentions of these entities (PROTEIN annotations) are further included as part of the input of the main tasks, the availability of these annotations could be assumed also in the REL task without introducing additional requirements on the task input. However, in the REL task setting, only one participant in each binary relation is a gene/protein NE, while the other can be either a non-name reference such as *promoter *or the name of an entity not of the gene/protein type (e.g. a protein complex). Motivated in part by the design goal to avoid introducing additional assumptions on input and the relatively limited number of existing methods for the detection of such entity references, their detection is included in the task: participants must recognize these secondary entities in addition to extracting the relations they participate in. These entities are assigned the generic type ENTITY.

**Figure 6:**
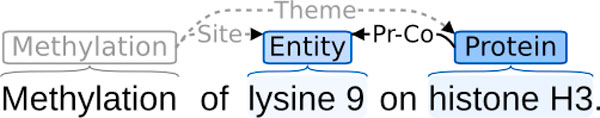
**Example REL relation annotation**. Simple REL annotation example showing a PROTEIN-COMPONENT (PR-CO) relation between "histone H3" and "lysine 9". An associated METHYLATION event and its arguments (shaded, not part of the REL task targets) shown for context.

The general task setting encompasses a rich set of potential relation extraction targets. For REL, we aimed to select relations that minimize overlap between the targets of other BioNLP ST'11 tasks while maintaining relevance as a supporting goal. As the main tasks primarily target events ("things that happen") involving change in entities, we chose to focus in the REL task on what we have previously termed "static relations" [67], that is, relations such as part-of that hold between entities without necessary implication of causality or change. A previous study further indicated that this class of relations may benefit event extraction [69]. We based our choice of specific target relations on previous studies of entity relations domain texts [67,68], which indicated that part-whole relations are by far the most frequent class of relevant relations for the task setting and proposed a classification of these relations for biomedical entities. We further found that - in terms of the taxonomy of Winston et al. [70] - object-component and collection-member relations account for the great majority of part-of relations relevant to the domain. For REL, we chose to omit collection-member relations in part to minimize overlap with the targets of the coreference task. Instead, we focused on two specific types of object-component relations, that holding between a gene or protein and its part (domains, regions, promoters, amino acids, etc.) and that between a protein and a complex that it is a subunit of. Following the biological motivation and the general practice in the shared task to term genes and gene products PROTEIN for simplicity, we named these two relations PROTEIN-COMPONENT and SUBUNIT-COMPLEX. Figure 6 shows an illustration of a simple relation with an associated event (not part of REL). Events with *Site *arguments such as that shown in the figure are targeted in the GE, EPI, and ID tasks that REL is intended to support.

### Corpora

Each of the three tasks makes use of manual annotations created specifically for the shared task, either entirely or in primary part. This section presents the three corpus resources on which these tasks are based.

#### EPI corpus

The primary EPI task data were annotated specifically for the BioNLP Shared Task 2011 and are not based on any previously released resource. Before starting this annotation effort, we performed two preparatory studies using related, previously released datasets: one considering the extraction of four protein post-translational modification event types [8], with reference to annotations originally created for the Protein Information Resource (PIR) [71,72], and one studying the annotation and extraction of DNA methylation events [10], with reference to annotations created for the PubMeth [73,74] database. The EPI corpus text selection and annotation scheme were then defined following the understanding formed in these studies.

#### EPI document selection

The texts for the EPI task corpus were drawn from PubMed abstracts. In selecting the primary corpus texts, we aimed to gather a representative sample of all PubMed documents relevant to selected modification events, avoiding bias toward, for example, specific genes/proteins, species, forms of event expression, or subdomains. We primarily targeted DNA methylation and the "prominent PTM types" identified in [8]. We defined the following document selection protocol: for each of the targeted event types,

1. Select a random sample of PubMed abstracts annotated with the MeSH term corresponding to the target event (e.g. Acetylation)

2. Automatically tag protein/gene entity mentions in the selected abstracts, removing abstracts where fewer than a specific cutoff are found

3. Perform manual filtering removing documents not relevant to the targeted topic (optional).

MeSH is a controlled vocabulary of over 25,000 terms that is used to manually annotate each document in PubMed. By performing initial document retrieval using MeSH terms it is possible to select relevant documents without bias toward specific expressions in text. While search for documents tagged with e.g. the Acetylation MeSH term is sufficient to select documents relevant to the modification, not all such documents necessarily concern specifically protein modification, necessitating a filtering step. Following preliminary experiments, we chose to apply the BANNER named entity tagger [59] trained on the GENETAG corpus [75] and to filter documents where fewer than five entities were identified. Finally, for some modification types this protocol selected also a substantial number of non-relevant documents. In these cases a manual filtering step was performed prior to full annotation to avoid creating detailed annotation for large numbers of non-relevant abstracts.

This primary corpus text selection protocol does not explicitly target reverse reactions such as deacetylation, and the total number of these events in the resulting corpus was low for many types. To be able to measure the extraction performance for these types, we defined a secondary selection protocol that augmented the primary protocol with a regular expression-based filter removing documents that did not (likely) contain mentions of reverse reactions. This protocol was used to select a secondary set of test abstracts enriched in mentions of reverse reactions. Performance on this secondary test set was also evaluated, but is not part of the primary task evaluation. Results on the secondary test set are reported to provide additional perspective the performance of systems at the EPI task.

#### EPI annotation

Annotation was performed manually. The gene/protein entities automatically detected in the document selection step were provided to annotators for reference for creating PROTEIN annotations, but all entity annotations were checked and revised to conform to the specific guidelines for the task (Due to differences in annotation criteria [76], substantial annotation was required). For the annotation of PROTEIN entities, we adopted the GENIA gene/gene product (GGP) annotation guidelines [77], adding one specific exception: while the primary guidelines require that only specific individual gene or gene product names are annotated, we allowed also the annotation of mentions of histone protein families or the entire histone superfamily to capture histone modification events also in cases where only the family is mentioned.

All event annotations were created from scratch without automatic support to avoid bias toward specific automatic extraction methods or approaches. The event annotation follows the GENIA event corpus annotation guidelines [78] as they apply to protein modifications, with CATALYSIS being annotated following the criteria for the POSITIVE REGULATION event type, with the additional constraints that the *Cause *of the event is a PROTEIN entity and the form of regulation is catalysis of a modification reaction. The manual annotation was performed by three experienced annotators with a molecular biology background. Annotator training and the overall annotation process was organized and supervised by an annotator with extensive experience in domain event annotation (TO).

After completion of primary annotation, we performed a final check targeting simple human errors using an automatic extraction system as follows: High-confidence system predictions differing from gold annotations were provided to a human annotator for re-evaluation (not used directly to change corpus data). To further reduce the risk of bias, we only informed the annotator of the entities involved, not of the predicted event structure. This correction process resulted in the revision of approximately 2% of the event annotations. To evaluate the consistency of the annotation, we performed independent event annotation (taking PROTEIN annotations as given) for a random sample of 10% of the corpus documents. Comparison of the two manually created sets of event annotations under the primary task evaluation criteria gave an F-score of 82% for the full task and 89% for the core task (due to symmetry of precision/recall and the applied criteria, this score was not affected by the choice of which set of annotations to consider as "gold" for the comparison). We found that CATALYSIS events were particularly challenging, showing just 65% agreement for the core task.

Table 3 shows the statistics of the primary EPI task data. We note that while the corpus is broadly comparable in size to the BioNLP ST'09 dataset [11] in terms of the number of abstracts and annotated entities, the number of annotated events in the EPI corpus is approximately 20% of that in the ST'09 dataset, reflecting the more focused event types.

**Table 3:** Statistics of the EPI corpus

Item	Train	Devel	Test	Total
Abstract	600	200	400	1,200
Word	127,312	43,497	82,819	253,628
Protein	7,595	2,499	5,096	15,190
Event	1,852	601	1,261	3,714
Modification	173	79	117	369

Test set statistics shown only for the primary test data.

#### ID corpus

The ID task data were newly annotated for the BioNLP Shared Task and are not based on any previously released resource. Annotation was performed by two teams, one in Tsujii laboratory (University of Tokyo) and one in Virginia Bioinformatics Institute (Virginia Tech). The entity and event annotation design was guided by previous studies on NER and event extraction in a closely related domain [79,80].

#### ID document selection

The training and test data were drawn from the primary text content of recent full-text PMC open access documents selected by infectious diseases domain experts (Virginia Tech team) as representative publications on two-component regulatory systems. Table 4 presents some characteristics of the corpus composition. To focus efforts on natural language text likely to express novel information, we excluded tables, figures and their captions, as well as methods sections, acknowledgments, authors' contributions, and similar meta-content.

**Table 4:** ID corpus composition

Journal	#	Published
PLoS Pathogens	9	2006-2010
PLoS One	7	2008-2010
BMC Genomics	3	2008-2010
PLoS Genetics	2	2007-2010
Open Microbiology J.	2	2008-2010
BMC Microbiology	2	2008-2009

Other	5	2007-2008

Journals in which selected articles were published with number of articles (#) and publication years.

#### ID annotation

Annotation was performed in two primary stages, one for marking core entities and the other for events and secondary entities. As a preliminary processing step, initial sentence segmentation was performed with the GENIA Sentence Splitter [81]. Segmentation errors were corrected during core entity annotation.

Core entity annotation was performed from the basis of an automatic annotation created using selected existing taggers for the target entities. The following tools and settings were adopted, with parameters tuned on initial annotation for two documents:

PROTEIN: NeMine [82] trained on the JNLPBA data [14] with threshold 0.05, filtered to only GENE and Protein types.

ORGANISM: Linnaeus [83] with "variant matching" for species names variants.

CHEMICAL: OSCAR3 [84] with confidence 90%.

TWO-COMPONENT-SYSTEM: Custom regular expressions.

Initial automatic tagging was not applied for entities of the REGULON-OPERON type or the generic ENTITY type (for additional event arguments). All automatically generated annotations were at least confirmed through manual inspection, and the majority of the automatic annotations were revised in manual annotation. Table 5 summarizes the tagging performance of the automatic tools as measured against the final human-annotated training and development datasets. It should be noted that these results are low in part due to differences in annotation criteria (see e.g. [76]) and to data tagged using the ID task annotation guidelines not being applied for training; training on the newly annotated data is expected to allow notably more accurate tagging.

**Table 5:** Automatic tagging performance for ID core entities

Entity type	**prec**.	**rec**.	F
PROTEIN	54.64	39.64	45.95
CHEMICAL	32.24	19.05	23.95
ORGANISM	90.38	47.70	62.44
TWO-COMPONENT-SYSTEM	87.69	47.24	61.40

Annotation for the task extraction targets - events and event modifications - was created entirely manually without automatic annotation support to avoid any possible bias toward specific extraction methods or approaches. The Tsujii laboratory team organized the annotation effort, with a coordinating annotator with extensive experience in event annotation leading annotator training and annotation scheme development, similarly as for the EPI corpus annotation. Detailed annotation guidelines [85] extending on the GENIA annotation guidelines [78] were developed jointly with all annotators and refined throughout the annotation effort. Based on measurements of inter-annotator consistency between annotations independently created by the two teams, made throughout annotator training and primary annotation (excluding final corpus cleanup), we estimate the consistency of the final entity annotation to be no lower than 90% F-score and that of the event annotation to be no lower than 75% F-score for the primary evaluation criteria (see the section on Evaluation).

#### ID datasets and statistics

Initial annotation was produced for the selected sections in 33 full-text articles, of which 30 were selected for the final dataset as representative of the extraction targets. These documents were split into training, development and test sets of 15, 5 and 10 documents, respectively. Participants were provided with all training and development set annotations and test set core entity annotations. The overall statistics of the datasets are given in Table 6.

**Table 6:** Statistics of the ID corpus

Item	Train	Devel	Test	Total
Article	15	5	10	30
Sentence	2,484	709	1,925	5,118
Word	74,439	21,225	57,489	153,153
Core entity	6,525	1,976	4,239	12,740
Event	2,088	691	1,371	4,150
Modification	95	45	74	214

As the corpus consists of full-text documents, it contains a somewhat limited number of articles, but in other terms it is of broadly comparable size to the largest of the BioNLP ST corpora: the corpus word count, for example, corresponds to that of a corpus of approximately 800 PubMed abstracts, and the core entity count is comparable to that in the ST'09 data. However, for reasons that may relate in part to the domain, the event count is approximately a third of that for the ST'09 data. In addition to having less training data, the entity/event ratio is thus considerably higher (i.e. there are more candidates for each true target), suggesting that the ID data could be expected to provide a more challenging extraction task.

#### REL corpus

The REL task dataset consists of new annotations for the GENIA corpus [6], building on the existing biomedical term annotation [27], the gene and gene product name annotation [77] and the syntactic annotation [86] of the corpus. The general features of the annotation are presented in [67], describing a previous release of a subset of the data. The REL task annotation effort extended the coverage of the previously released annotation to all relations of the targeted types stated within sentence scope in the GENIA corpus.

For compatibility with the ST'09 and the ST'11 GE task, the REL task training/development/test set division of the GENIA corpus abstracts matches that of the ST'09 data. The statistics of the corpus are presented in Table 7. We note that both in terms of training examples and the data available in the given development set, the number of examples of the PROTEIN-COMPONENT relation is more than twice that for SUBUNIT-COMPLEX. Thus, at least for methods based on machine learning, we might generally expect to find higher extraction performance for the former relation.

**Table 7:** Statistics of the REL corpus

Item	Train	Devel	Test	Total
Abstract	800	150	260	1,210
Word	176,146	33,827	57,256	267,229
Protein	9,297	2,080	3,589	14,966

Relation	1,857	480	497	2,834
PROTEIN-COMPONENT	1,302	314	334	1,950
SUBUNIT-COMPLEX	555	166	163	884

### Evaluation

Evaluation for all tasks is based on comparison of submissions from participants against gold standard data prepared in advance for each task. Performance measurement is instance-oriented - that is, each individual annotation (event or relation) is considered separately - and based on the standard precision, recall and F-score metrics. Specifically, *F*_1_, the harmonic mean of precision and recall, is used to combine precision and recall and used as the primary ranking criterion in all tasks. The *F*_1 _score is referred to as F-score for short throughout.

The primary evaluation criteria for determining whether a predicted event matches a gold event in the EPI and ID tasks are the same as in the BioNLP ST'09 and the GE task. These criteria relax exact matching in two aspects, incorporating *approximate span matching *and *approximate recursive matching*. Under approximate span matching, text-bound annotations (event triggers and ENTITY type entities) in a submission are considered to match a corresponding gold annotation if their span is contained within the expansion of the gold span by one word to both the left and the right. Under approximate recursive matching, events that refer to other events as arguments are considered to match if the *Theme *arguments of the recursively referred events match, that is, non-*Theme *arguments are ignored for recursively referred events. For an extended discussion of these evaluation criteria, we refer to their definition in the BioNLP ST'09 overview [11].

In addition to the primary evaluation criteria, we consider a new relaxed event evaluation criterion that we term *single partial penalty*. Under the primary criteria, when a predicted event matches a gold event in some of its arguments but lacks one or more arguments of the gold event, the submission is arguably given a double penalty: the predicted event is counted as a false positive (FP), and the gold event is counted as a false negative (FN). Under the single partial penalty evaluation criterion, predicted events that match a gold event in all their arguments but do not contain all the arguments of the gold event are not counted as FP, although the corresponding gold event still counts as FN (the "single penalty"). Analogously, gold events that partially match a predicted event are not counted as FN, although the corresponding predicted event with "extra" arguments counts as FP. This criterion is intended to provide a more nuanced view of performance for partially correctly predicted events.

The full ID and EPI tasks involve many partially independent challenges, requiring the extraction of all event arguments (both *core *and *additional*) as well as the detection of event modifications (NEGATION and SPECULATION). These aspects of event extraction are treated as separate subtasks in the BioNLP ST'09 and the GE task, where the identification of additional event arguments is subtask 2 and the detection of negated and speculated events subtask 3. While explicit subtasks are not defined for ID and EPI, both tasks specify in addition to the *full *task targets also minimal *core *extraction targets, consisting of events with only their core arguments and excluding event modifications. Results are then reported for each submission separately for evaluation against the full gold standard data (full task) and for evaluation where both the gold standard data and the submission events are reduced to only core arguments, event modifications are removed, and resulting duplicate events removed (core task). In terms of the subtask structure of the BioNLP ST'09 and the GE task, the core task is analogous to subtask 1 and the full task analogous to the combination of subtasks 1-3.

The evaluation of the REL task relations parallels the criteria for event evaluation in the main tasks. The REL task also relaxes the equality criteria for matching text-bound annotations: for a submission entity to match an entity in the gold reference annotation, it is sufficient that the span of the submitted entity (i.e. its start and end positions in text) is entirely contained within the span of the gold annotation. This corresponds largely to the approximate span matching criterion for events, although the REL criterion is slightly stricter in not involving testing against an extension of the gold entity span. Relation matching is exact: for a submitted relation to match a gold one, both its type and the related entities must match.

The statistical significance of the differences in system performance is evaluated using the approximate randomization method with 9,999 repetitions [87,88].

## Results

### Participation

Final results to each of the EPI and ID main tasks were successfully submitted by seven participants and results for the REL supporting task by four participants.

Table 8 summarizes the groups participating in one or more of these tasks, and Table 9 the features of the event and relation extraction systems. We note that, similarly to the ST'09 task, machine learning-based systems remain dominant overall, although there is considerable divergence in the specific methods applied. In addition to domain mainstays such as support vector machines and maximum entropy models, we find increased application of joint models [89-91] as opposed to pure pipeline systems [92-94]. Remarkably, the application of full parsing is adopted in all systems and the use of dependency-based representations of syntactic analyses in all but one. Further, there is significant uniformity in the specific choice of tools for syntactic analysis: the parser of Charniak and Johnson [95] with the biomedical domain model of McClosky [96] and conversion into the Stanford Dependency representation [97] is applied in six out of nine event extraction systems and all but one relation extraction system. These choices may be motivated in part by the success of systems using the tools in the previous shared task and the availability of the analyses as supporting resources [98].

**Table 8:** Teams, ranks and system descriptions

Team	Tasks (rank)	Organization	System descriptions
FAUST	EPI (2), ID (1)	3 NLP researchers	Riedel *et al*. [90], McClosky *et al*. [108]
UTurku	EPI (1), ID (5), REL(1)	1 bioinformatician	Björne *et al*. [93,109,110]
VIBGhent	REL(2)	1 NLP and 1 machine learning researcher, 1 bioinformatician	Van Landeghem *et al*. [101,111]
UMass	EPI (4), ID (2)	1 NLP researcher	Riedel *et al*. [112], Riedel and McCallum [92,113]
MSR-NLP	EPI (3)	1 software engineer, 3 NLP researchers	Quirk *et al*. [94]
Stanford	EPI (5), ID (3)	3 NLP researchers	McClosky *et al*. [91,114]
ConcordU	EPI (7), ID (4), REL(3)	2 NLP researchers	Kilicoglu and Bergler [100,115,116]
CCP-BTMG	EPI (6)	3 bioinformaticians	Liu *et al*. [117]
PNNL	ID (6)	1 computer scientist, 1 NLP researcher, 2 bioinformaticians	McGrath *et al*. [95]
PredX	ID (7)	1 computer scientist, 1 NLP researcher	-
HCMUS	REL (4)	6 linguists	Quang *et al*. [102]

Profiles of teams, tasks participated in (with ranks), and references to published system descriptions.

**Table 9:** Summary of extraction system architectures

Event extraction systems							
	NLP		Events				
Team	Word	Parse	Trig.	Arg.	Group.	Modif.	Other resources
FAUST	CoreNLP, SnowBall	McCCJ + SD	(UMass+Stanford as features)			-	word clusters
UTurku	Porter	McCCJ + SD	SVM	SVM	SVM	SVM	hedge words
UMass	CoreNLP, SnowBall	McCCJ + SD	Joint, dual decomposition			-	-
MSR-NLP	Porter, custom	McCCJ + SD, Enju	SVM	SVM	SVM	-	triggers, word clusters
Stanford	custom	McCCJ + SD	MaxEnt		Joint, MSTParser	-	word clusters
ConcordU	-	McCCJ + SD	Dict	Rules	Rules	Rules	triggers and hedge words
CCP-BTMG	Porter, WN-lemma	Stanford + SD	Graph extraction & matching			-	-
PNNL	Porter	Stanford	SVM	SVM	Rules	-	UMLS, triggers
PredX	LGP	LGP	Dict	Rules	Rules	-	UMLS, triggers
**Relation extraction systems**							
	**NLP**		**Extraction**		**Other resources**		
**Team**	**Word**	**Parse**	**Entities**	**Relations**	**Corpora**	**Other**	
UTurku	Porter	McCCJ + SD	SVM	SVM	-	-	
VIBGhent	Porter	McCCJ + SD	Dict + rules	SVM	GENIA, PubMed	word similarities	
ConcordU	-	McCCJ + SD	Dict	Rules	-	-	
HCMUS	OpenNLP	OpenNLP	Dict	Rules	-	-	

Abbreviations: Trig./Arg./Group./Modif.=event trigger detection/argument detection/argument grouping/modification detection, CoreNLP = Stanford CoreNLP [117], Porter=Porter stemmer [118], Snowball=Snowball stemmer [119], WN-lemma=WordNet lemmatization, McCCJ=McClosky-Charniak-Johnson parser [97,98], LGP = Link Grammar Parser [120], SD=Stanford Dependency conversion [97], Dict=Dictionary, UMLS=UMLS resources (e.g. lexicon, metamap) [121].

Several participants compiled dictionaries of event trigger words and two dictionaries of hedge words from the data. The use of dictionary-based approaches instead of machine learning for these components may reflect challenges in training with the trigger and hedge annotations. Trigger annotation is not exhaustive as triggers are not included in gold data e.g. for event statements where the participants fall out of scope of the entity annotation, and negation and speculation are annotated without specific trigger/cue words, precluding direct training for modifier cue detection. Despite the existence of rich lexical resources in the domain, only two groups applied databases such as UMLS in their systems. The results indicate that such resources are not critical for success at the task, a somewhat surprising result that may merit further investigation. In addition to manually curated external resources, ones derived from large-scale unannotated data through unsupervised methods were used by three groups for event extraction and one for relation extraction.

Table 10 summarizes the use of corpus resources other than the task training data in the main tasks. Despite the availability of PTM and DNA methylation resources other than those specifically introduced for the task and the PHOSPHORYLATION annotations included in the GE corpus [54], no participant chose to apply other corpora for training in the EPI task. With the exception of externally acquired unlabeled data, the EPI task results thus reflect a closed task setting in which only the given data is used for training. By contrast, in the ID task four teams - including the three top-ranking - used the GE task corpus as supplementary material. The findings indicate that the GE corpus, containing approximately three times as many event annotations as ID, is largely compatible with the ID annotations and can be beneficially combined with the smaller in-domain corpus (see detailed results below). This result is encouraging for future applications of the event extraction approach: as manual annotation requires considerable effort and time, the ability to use large existing annotated resources is important for the feasibility of adaptation of the approach to new domains.

**Table 10:** Summary of use of corpus resources

	EPI		ID	
Team	Rank	Corpora	Rank	Corpora
FAUST	2	-	1	GE
UTurku	1	-	5	-
UMass	4	-	2	GE
MSR-NLP	3	-	N/A	
Stanford	5	-	3	GE
ConcordU	7	-	4	-
CCP-BTMG	6	-	N/A	
PNNL	N/A		6	GE
PredX	N/A		7	-

Use of corpora other than the task training and development data in the EPI and ID main tasks.

While several participants made use of supporting syntactic analyses provided by the organizers [98], none applied the analyses for supporting tasks such as coreference or REL - at least in cases due to time constraints [99]. There may thus remain further opportunities for improvement through combinations of supporting analyses with main task extraction systems.

We find a remarkable number of similarities between the approaches also for the REL supporting task, with all four utilizing full parsing and a dependency representation of the syntactic analysis, and the three highest-ranking further using the same parser and specific dependency representation. While UTurku [92] and VIBGhent [100] further agree in the choice of Support Vector Machines for the extraction of relations, ConcordU [99] and HCMUS [101] pursue approaches building on dictionary- and rule-based extraction. Only the VIBGhent system makes use of resources external to those provided for the task, extracting specific semantic entity types from the GENIA corpus as well as inducing word similarities from a large unannotated corpus of PubMed abstracts.

### EPI primary evaluation results

**Figure 7:**
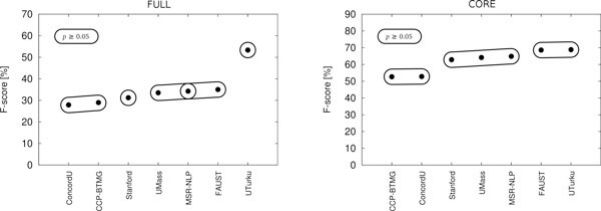
**Statistical significance of EPI task results**. Results for full task on the left, core task on the right. Enclosed areas identify sets of systems for which performance differences were not statistically significant; for systems not sharing an enclosed area, differences are significant (*p *< 0.05).

Table 11 presents the EPI task primary results by event type, Table 12 (left) summarizes these results, and Figure 7 (left) illustrates the results of the evaluation of the statistical significance of their differences.

**Table 11:** EPI task primary evaluation results: F-scores by event type

	UTurku	FAUST	MSR- NLP	UMass	Stanford	CCP- BTMG	ConcordU	Size
HYDROXYLATION	**42.25**	10.26	10.20	12.80	9.45	12.84	6.32	139
DEHYDROXYLATION	-	-	-	-	-	-	-	1
PHOSPHORYLATION	**67.12**	51.61	50.00	49.18	40.98	47.06	44.44	130
DEPHOSPHORYLATION	0.00	0.00	0.00	0.00	0.00	**50.00**	0.00	3
UBIQUITINATION	**75.34**	72.95	67.88	72.94	67.44	70.87	69.97	340
DEUBIQUITINATION	**54.55**	40.00	0.00	31.58	0.00	42.11	14.29	17
DNA METHYLATION	**60.21**	31.21	34.54	23.82	31.02	15.65	8.22	416
DNA DEMETHYLATION	**26.67**	0.00	0.00	0.00	0.00	0.00	0.00	21
*Simple event total*	***63*.*05***	*45.17*	*44.97*	*43.01*	*40.96*	*40.62*	*37.84*	*1067*
GLYCOSYLATION	**49.43**	41.10	38.87	40.00	37.22	25.62	25.94	347
DEGLYCOSYLATION	**40.00**	35.29	0.00	38.10	30.00	35.29	26.67	27
ACETYLATION	**57.22**	40.00	41.42	40.25	35.12	37.50	38.19	337
DEACETYLATION	**54.90**	28.00	31.82	29.17	21.74	24.56	27.27	50
METHYLATION	**57.67**	24.82	19.57	23.67	18.54	16.99	15.50	374
DEMETHYLATION	**35.71**	0.00	0.00	0.00	0.00	0.00	0.00	13
*Non-simple event total*	***54.36***	*33.86*	*31.85*	*33.07*	*29.28*	*25.06*	*25.10*	*1148*
CATALYSIS	**7.06**	6.58	**7.75**	5.00	2.84	7.58	1.74	238
*Subtotal*	***55.02***	*36.93*	*36.17*	*35.30*	*32.85*	*30.58*	*28.92*	*2453*

NEGATION	18.60	0.00	0.00	0.00	0.00	0.00	**26.51**	149
SPECULATION	**37.65**	0.00	0.00	0.00	0.00	0.00	6.82	103
*Modification total*	***28.07***	*0.00*	*0.00*	*0.00*	*0.00*	*0.00*	*16.37*	*252*

*Total*	***53.33***	*35.03*	*34.27*	*33.52*	*31.22*	*28.97*	*27.88*	*2705*

*Addition total*	***59.33***	*40.27*	*39.05*	*38.65*	*36.03*	*32.75*	*31.50*	*2038*

*Removal total*	***44.29***	*22.41*	*15.73*	*22.76*	*14.41*	*23.53*	*17.48*	*132*

The Size column gives the number of annotations of each type in the given data (training+development). Best result for each type shown in bold. For DEHYDROXYLATION, no examples were present in the test data and none were predicted by any participant.

**Table 12:** EPI task evaluation results: summary

	Primary (full)			Core			
Team	recall	**prec**.	F-score	recall	**prec**.	F-score	Δ_*f*_
UTurku	52.69	53.98	53.33	68.51	69.20	68.86	15.53
FAUST	28.88	44.51	35.03	59.88	80.25	68.59	33.56
MSR-NLP	27.79	44.69	34.27	55.70	77.60	64.85	30.58
UMass	28.08	41.55	33.52	57.04	73.30	64.15	30.63
Stanford	26.56	37.85	31.22	56.87	70.22	62.84	31.62
CCP-BTMG	23.44	37.93	28.97	40.28	76.71	52.83	24.95
ConcordU	20.83	42.14	27.88	45.06	63.37	52.67	23.70

The Δ*_f _*column gives the F-score difference of each core task result to the corresponding primary result.

We note that only two teams, UTurku [93] and ConcordU [100], predicted event modifications, and only UTurku predicted additional (non-core) event arguments (data not shown). The other five systems thus addressed only the core task. For the full task, this difference in approach is reflected in the substantial performance advantage for the UTurku system, which exhibits highest performance overall as well as for most individual event types. The results suggest that the ability to recover additional arguments is key to competitive performance on the EPI full task.

Extraction performance for simple events taking only *Theme *and *Site *arguments is consistently higher than for other event types, with absolute F-score differences of over 10% points for many systems. Similar notable performance differences are seen between the *addition *events, for which ample training data was available, and the *removal *types for which data was limited (Size column in Table 11). This effect is particularly noticeable for DEPHOSPHORYLATION, DNA DEMETHYLATION and DEMETHYLATION, for which the clear majority of systems failed to predict any correct events.

Extraction performance for CATALYSIS events is very low despite a relatively large set of training examples, indicating that the extraction of nested event structures remains very challenging. This low performance may also be related to the fact that CATALYSIS events are often triggered by the same word as the catalysed modification (e.g. Figure 1b), requiring the assignment of multiple event labels to a single word in typical system architectures.

Table 12 (right) summarizes the EPI core task results and and Figure 7 (right) illustrates the statistical significance of the differences. While all systems show notably higher performance than for the full task, high-ranking participants focusing on the core task gain most dramatically, with the FAUST system core task F-score essentially matching that of the top system (UTurku). For the core task, all participants achieve F-scores over 50% - a level of performance achieved by only a single system in the ST'09 task - and the top four participants average over 65% F-score. These results confirm that current event extraction technology is well applicable to the core PTM extraction task, even when the number of targeted event types is relatively high, and may be ready to address the challenges of exhaustive PTM extraction [9]. The best core tasks results, approaching 70% F-score, are particularly encouraging as the level of performance is comparable to or better than state-of-the-art results for many reference resources for protein-protein interaction extraction (see e.g. [102]) using the simple untyped entity pair representation, a standard task that has been extensively studied in the domain.

### ID primary evaluation results

Table 13 presents the primary results of the ID task by event type, and Table 14 (left) summarizes these results. Evaluation of statistical significance showed that the differences in overall performance are significant for all pairs of systems (*p *< 0.05).

**Table 13:** ID task primary evaluation results: F-scores by event type

	FAUST	UMass	Stanford	ConcordU	UTurku	PNNL	PredX	Size
GENE EXPRESSION	**70.68**	66.43	54.00	56.57	64.88	53.33	0.00	512
TRANSCRIPTION	69.66	68.24	60.00	**70.89**	57.14	0.00	53.85	77
PROTEIN CATABOLISM	**75.00**	72.73	20.00	66.67	33.33	11.76	0.00	33
PHOSPHORYLATION	64.00	**66.67**	40.00	54.55	60.61	64.29	40.00	69
LOCALIZATION	33.33	14.29	31.58	20.00	**66.67**	20.69	0.00	49
*Simple event total*	***68.47***	*63.55*	*52.72*	*56.78*	*62.67*	*43.87*	*18.18*	*740*
BINDING	31.30	34.62	23.44	**40.00**	22.22	20.00	28.28	156
PROCESS	65.69	62.26	**73.57**	67.17	41.57	51.04	53.27	901
*Non-regulation total*	***63.78***	*60.68*	*63.59*	*62.43*	*46.39*	*47.34*	*43.65*	*1797*

REGULATION	**35.44**	30.49	17.67	19.43	22.96	0.00	2.16	267
POSITIVE REGULATION	47.50	**49.49**	34.78	23.41	41.28	24.60	21.02	455
NEGATIVE REGULATION	58.86	**60.45**	44.44	47.96	52.11	25.70	9.49	260
*Regulation total*	***47.07***	*46.65*	*33.02*	*28.87*	*39.49*	*18.45*	*9.71*	*982*

*Subtotal*	***57.28***	*55.03*	*52.09*	*46.60*	*43.33*	*37.53*	*28.38*	*2779*

NEGATION	0.00	0.00	0.00	22.92	**32.91**	0.00	0.00	96
SPECULATION	0.00	0.00	0.00	3.23	**15.00**	0.00	0.00	44
*Modification total*	0.00	0.00	0.00	11.82	**26.89**	0.00	0.00	140

*Total*	***55.59***	*53.42*	*50.63*	*44.21*	*42.57*	*36.27*	*27.49*	*2919*

The Size column gives the number of annotations of each type in the given data (training+development). Best result for each type shown in bold.

**Table 14:** ID task evaluation results: summary

	Primary (full)			Core			
Team	recall	**prec**.	F-score	recall	**prec**.	F-score	Δ_*f*_
FAUST	48.03	65.97	55.59	50.84	66.35	57.57	1.98
UMass	46.92	62.02	53.42	49.67	62.39	55.31	1.89
Stanford	46.30	55.86	50.63	49.16	56.37	52.52	1.89
ConcordU	49.00	40.27	44.21	50.91	43.37	46.84	2.63
UTurku	37.85	48.62	42.57	39.23	49.91	43.93	1.36
PNNL	27.75	52.36	36.27	29.36	52.62	37.69	1.42
PredX	22.56	35.18	27.49	23.67	35.18	28.30	0.81

The full ID task requires the extraction of additional arguments and event modifications and involves multiple novel challenges from previously addressed event extraction tasks including a new subdomain, full-text documents, several new entity types and a new event category. Nevertheless, extraction performance for the top systems is comparable to the state-of-the-art results for the established BioNLP ST'09 task [103] as well as its repetition as the 2011 GE task [54], where the highest overall result for the primary evaluation criteria was also 56% F-score for the FAUST system [89]. This result is encouraging regarding the ability of the extraction approach and methods to generalize to new domains as well as their applicability specifically to texts on the molecular mechanisms of infectious diseases.

We note that there is substantial variation in the relative performance of systems for different entity types. For example, Stanford [89] has relatively low performance for simple events but achieves the highest result for PROCESS, while UTurku [92] results show roughly the reverse. This suggests further potential for improvement from system combinations.

The best performance for simple events and for PROCESS approaches or exceeds 70% F-score, arguably approaching a sufficient level for user-facing applications of the extraction technology. By contrast, BINDING and regulation events, found challenging in ST'09 and GE, remain problematic also in the ID task, with best overall performance below 50% F-score. As in the EPI task, only the UTurku and ConcordU [99] teams attempted to extract event modifications, with somewhat limited performance. The difficulty of correct extraction of event modifications is related in part to the recursive nature of the problem (similarly as for nested regulation events): to extract a modification correctly, the modified event must also be extracted correctly. Again as for EPI, only UTurku predicted any instances of secondary arguments. Thus, teams other than UTurku and ConcordU addressed only the core task extraction targets. However, by contrast to the EPI results, lacking prediction of additional arguments does not preclude systems from competitive performance at the ID task.

With the exception of ConcordU, all systems clearly favor precision over recall (Table 14, left), in many cases having over 15% point higher precision than recall. This a a somewhat unexpected inversion as the ConcordU system is rule-based, an approach typically associated with high precision.

The five top-ranking systems participated also in the GE task [54], which involves a subset of the ID extraction targets. This allows additional perspective into the relative performance of the systems. While there is a 13% point spread in overall results for the top five systems here, in GE all these systems achieved F-scores ranging between 50-56%. The results for FAUST, UMass and Stanford were similar in both tasks, while the ConcordU result was 6% points higher for GE and the UTurku result over 10% points higher for GE, ranking third after FAUST and UMass. These results suggest that while the FAUST and UMass systems in particular have some systematic (e.g. architectural) advantage at both tasks, much of the performance difference observed here between the top three systems and those of ConcordU and UTurku is due to strengths or weaknesses specific to ID. Possible weaknesses may relate to the treatment of multiple core entity types (vs. only PROTEIN in GE), challenges related to nested entity annotations (not appearing in GE), or the new PROCESS type. A possible ID-specific strength of the three top-ranking systems is the use of GE data for training: Riedel and McCallum [91] report an estimated 7% point improvement and McClosky et al. [90] a 3% point improvement from use of this data; McGrath et al. [94] estimate a 1% point improvement from direct corpus combination. The integration strategies applied in training these systems could potentially be applied also with other systems, an experiment that could further clarify the relative strengths of the various systems. The top-ranking five systems all participated also in the EPI task, for which UTurku ranked first with FAUST having comparable performance for the core task. While this supports the conclusion that ID performance differences do not reflect a simple universal ranking of the systems, due to many substantial differences between the ID and EPI setups it is not straightforward to identify specific reasons for relative differences to performance at EPI.

Table 14 (right) summarizes the ID core task results. Please note that these results differ (by up to 0.5% point in overall F-score) from those reported previously [31] due to the correction of an error in the original core task evaluation system. As for the primary ID results, all the differences in system performance for the core task are also statistically significant (*p *< 0.05). By contrast to the results for EPI, where differences of over 30% points were observed between full and core task results, there are only modest and largely consistent differences to the corresponding full task results for ID, reflecting in part the relative sparseness of additional arguments: in the training data, for example, only approximately 3% of instances of event types that can potentially take additional arguments had one or more additional arguments. While event modifications represent a further 4% of full task extraction targets not required for the core task, the overall low extraction performance for additional arguments and modifications limits the practical effect of these annotation categories on the performance difference between systems addressing only the core targets and those addressing the full task.

### REL evaluation results

Table 15 shows the results of the REL task. We find that the four systems diverge substantially in terms of overall performance, with all pairs of systems of neighboring ranks showing differences approaching or exceeding 10% points in F-score. While three of the systems notably favor precision over recall, VIBGhent shows a decided preference for recall, suggesting a different approach from UTurku in design details despite the similarities in relation extraction approach. The highest-performing system, UTurku, shows an F-score in the general range of state-of-the-art results in the main event extraction tasks, which could be taken as an indication that the reliability of REL task analyses created with presently available methods may not be high enough for direct use as a building block for the main tasks. However, the emphasis of the highest-scoring system on precision is encouraging for such applications: nearly 70% of the entity-relation pairs that the system predicts are correct. The two top-ranking systems show similar precision and recall results for the two relation types. The submission of HCMUS shows a decided advantage for PROTEIN-COMPONENT relation extraction as tentatively predicted from the relative numbers of training examples, but their rule-based approach suggests training data size is likely not the decisive factor. While the limited amount of data available prevents strong conclusions from being drawn, overall the lack of correlation between training data size and extraction performance suggests that performance may not be primarily limited by the size of the available training data.

**Table 15:** REL task primary evaluation results

	UTurku	VIBGhent	ConcordU		HCMUS	
PROTEIN-COMPONENT	50.90 / 68.57 / **58.43**	47.31 / 36.53 / 41.23	23.35 / 52.05 / 32.24	20.96	/ 21.63	/ 21.29
SUBUNIT-COMPLEX	48.47 / 66.95 / **56.23**	47.85 / 38.12 / 42.43	26.38 / 39.81 / 31.73	4.91	/ 66.67	/ 9.14

Total	50.10 / 68.04 / **57.71**	47.48 / 37.04 / 41.62	24.35 / 46.85 / 32.04	15.69	/ 23.26	/ 18.74

Results given as recall/precision/F-score.

### Additional evaluation results

Table 16 (left) summarizes the full EPI task results with the addition of the single partial penalty criterion. The F-scores for the seven participants under this criterion are on average over 4% points higher than under the primary criteria, with the most substantial increases seen for high-ranking participants only addressing the core task: for example, the precision of the FAUST system [89] is nearly 30% higher under the relaxed criterion. The core evaluation results showed that the limited precision of these systems for the full task is not due to errors in core argument recognition but rather due to lack of recognition for secondary arguments. From an application-oriented perspective it could be argued that the high precision but low recall seen for the single partial penalty criterion is a more accurate representation of actual system performance than seen under the primary criteria, as the systems make few outright false predictions, even though they do lack much of the detail of the full annotation. Despite substantial effects on precision in many cases, the overall F-score difference to the main criteria remains limited due to the low recall of many systems.

**Table 16:** Evaluation results with single partial penalty

	EPI				ID				
Team	recall	**prec**.	F-score	Δ*_f_*	Team	recall	**prec**.	F-score	Δ_*f*_
UTurku	54.79	58.42	56.55	3.22	FAUST	50.52	71.64	59.25	3.66
FAUST	28.88	72.05	41.24	6.21	UMass	49.00	68.21	57.03	3.61
MSR-NLP	27.79	66.72	39.24	4.97	Stanford	48.44	60.20	53.68	3.05
UMass	28.08	63.28	38.90	5.38	ConcordU	52.46	42.50	46.96	2.75
Stanford	26.56	56.83	36.20	4.98	UTurku	42.28	50.51	46.03	3.46
CCP-BTMG	23.44	50.79	32.08	3.11	PNNL	35.99	53.48	43.02	6.75
ConcordU	20.83	60.55	30.99	3.11	PredX	24.08	39.95	30.05	2.56

Results for EPI and ID full tasks, primary evaluation criteria with single partial penalty. The Δ_*f *_columns give F-score differences to the corresponding primary results.

Table 16 (right) shows the results for the ID task under the single partial penalty criterion. The average difference to F-score for primary criteria is broadly comparable to that for EPI, at slightly less than 4% points. However, this change is due to a very different effect on precision and recall: while for EPI a strong increase in evaluated precision was observed, for ID the increase is more evenly distributed and in cases observable as an increase in recall. This suggests that while in EPI missing arguments were a key limiting factor for performance in most cases, for ID there are comparatively many cases where systems make the error of predicting extra arguments that are not found in otherwise matching gold annotations. It is also interesting to note that while core evaluation is by far the most permissive setting for EPI, for ID higher F-scores are achieved in the full task with the single partial penalty criterion.

Evaluation using single partial penalty allows additional perspectives into both the absolute and the comparative performance of systems. The results also indicate possible directions for seeking more meaningful criteria for event extraction system evaluation. While there are no single "correct" answers, further basis for evaluating candidate answers can be provided through manual evaluation reflecting the perceived quality of system outputs, considered in the following.

Finally, Table 17 summarizes the results for the EPI extra test set enriched for events that were relatively sparse in the training data; especially reverse reactions. While these results show the expected drop associated with sparse training data for some systems, surprisingly a reverse effect is seen for others, particularly clearly for UMass. This result is unexpected also as UMass performance for reverse reactions on the main test data was comparatively weak. The mixed results for this additional test data have not suggested to us any straightforward conclusions regarding the comparative strengths of the various approaches, and we will here refrain from speculation regarding possible explanations. Nevertheless, this additional test data remains available for further experiments and evaluation and can hopefully support detailed study of effective strategies for event extraction with limited training data.

**Table 17:** EPI task evaluation results for extra test set

Team	recall	**prec**.	F-score	Δ*_f_*
UTurku	38.46	57.87	46.21	-7.12
UMass	28.00	60.26	38.24	4.72
FAUST	24.62	53.33	33.68	-1.35
Stanford	23.69	53.10	32.77	1.55
MSR-NLP	22.46	46.79	30.35	-3.92
CCP-BTMG	22.15	47.06	30.13	1.16
ConcordU	12.92	41.58	19.72	-8.16

Results using primary criteria. The Δ*_f _*column gives differences to the main test set results for the same criteria. Results for this data are not considered part of the primary evaluation for the task.

### System combination

The event extraction system analyses created by the participants represent a rich resource for further study of the task. To explore one opportunity presented by this data, we studied whether the outputs of various systems could be beneficially combined to further improve extraction performance. We performed a straightforward combination of the output of all systems participating in the EPI and ID main tasks using a strategy where the combination system only outputs an event if it is found in the output of at least *n *combined systems, where *n *is a parameter ranging between one and the total number of systems. For determining event equality for the combination, we applied the primary evaluation metrics (approximate span and recursive matching). As the majority of systems only addressed the core tasks, we measured the performance of system combinations only on the core extraction targets.

**Figure 8:**
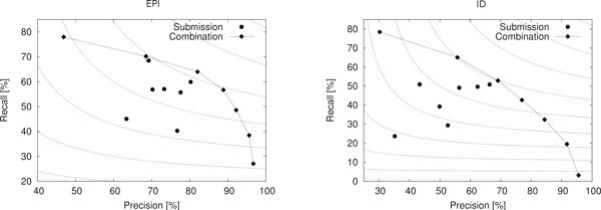
**Performance of submissions and system combinations for the core tasks**. Results for EPI on the left, ID on the right. Contours correspond to F-score levels of (10, 20, . . .).

Table 18 (left) shows the results of the combination experiment for the core EPI task and Figure 8 (left) plots the performance of the seven submissions and the seven combinations. We find that for *n *∈ (2, 3, 4) the combinations outperform the highest standalone result for the core task (68.86%) in terms of F-score, improving on the standalone result by over 3% points in F-score for *n *= 3, thus providing an approximately 10% relative decrease in error.

**Table 18:** Core task evaluation results for system combinations

	EPI			ID			
*n*	recall	**prec**.	F-score	*n*	recall	**prec**.	F-score
1	77.97	46.88	58.55	1	78.45	30.25	43.67
2	70.18	68.52	69.34	2	65.08	55.79	60.08
3	63.99	82.06	71.91	3	52.89	69.08	59.91
4	56.70	88.85	69.22	4	42.66	76.94	54.89
5	48.58	92.21	63.63	5	32.43	84.41	46.86
6	38.44	95.62	54.84	6	19.50	91.75	32.17
7	27.05	96.71	42.28	7	3.14	95.56	6.08

Different choices of *n *in calculating the combination permit the choice of various points along a precision-recall curve spanning nearly the full precision range, giving nearly 97% precision for *n *= 7. However, no combination permits for comparably high values of recall: even for *n *= 1, corresponding to the union of all submissions, recall remains below 80%. There thus remains a substantial number of events that none of the systems can retrieve.

Table 18 (right) and Figure 8 (right) present the system combination results for the ID task. Also for ID the system combination outperforms the best standalone result (57.57% F-score) for *n *= 2 and *n *= 3. The advantage is somewhat less than for EPI, approximately 2.5% points or an approximately 6% decrease in error. The more limited benefit from system combination for ID may be explained in part by the greater variance in the performance of individual systems for the task.

We note that the system combination strategies explored here are comparatively simple and make no use of information such as the relative reliability of the different systems [11], which could be separately estimated e.g. using the development data. There is a possibility that the performance of system combinations could be further substantially improved through the use of advanced combination strategies.

### Manual analysis

Evaluation against a fixed gold standard provides a stable, objective basis for the comparison of systems and, when feasible, is often the preferred choice for the study of new tasks such as those introduced in this study. A fixed, fully annotated gold standard has the further benefit of allowing the results of experiments performed after its original introduction to be directly compared against those of the original work on an even basis.

Nevertheless, comparison against gold standard annotation such as that applied in the BioNLP Shared Tasks also has its drawbacks when compared to alternatives such as direct evaluation of system outputs. One such drawback is the difficulty of assessing degrees of correctness for system outputs that differ from gold annotation. For any non-trivial representation aiming to capture (part of) the meaning of complex text there will almost unavoidably be cases where more than one set of annotations are at least acceptable; where the annotation task takes on aspects of *translation *from natural language into the semantic representation. Tightly specified annotation guidelines can (and in gold standard annotation arguably should) be used to rule out all but one candidate interpretation (cf. controlled language). However, from the perspective of many applications such constraints do not change the basic issue that declaring one out of several potentially acceptable analyses "correct" and others "incorrect" can lead to a distorted picture of system (or human annotator) performance.

**Figure 9:**
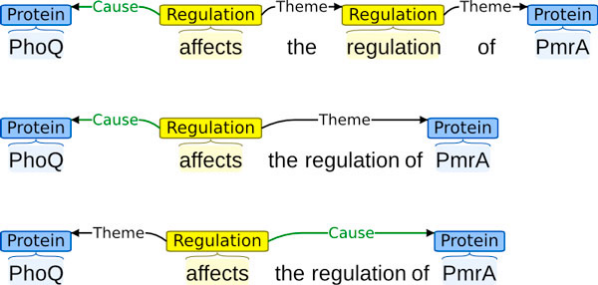
**Illustration of possible annotations for an example sentence**. Top: correct (by guideline), middle: acceptable, bottom: incorrect.

To take a specific example, the annotation guidelines of the GE and ID tasks require each "link" in a stated chain of causation to be separately marked, so that e.g. *A affects the regulation of B *is marked with two REGULATION annotations (Figure 9 top). However, from the perspective of many applications (e.g. pathway curation and semantic search), an annotation involving a single REGULATION event correctly identifying the direction of causality (Figure 9 middle) may be equally acceptable, or at least clearly preferable to an annotation where the direction is inverted (Figure 9 bottom). Yet in evaluation against the gold standard the latter two are both equally wrong.

Another drawback of the evaluation against a gold standard is that errors in gold annotation - unavoidable in any human product of this complexity - will tend to bias results against the systems, leading to underestimates of performance. The tendency of gold annotation errors to hurt rather than help systems in evaluation arises for any representation where there are many more ways to annotate wrongly than correctly, as the likelihood of an erroneous gold annotation matching an erroneous system output will be comparatively small.

To provide additional perspective to the performance of the systems participating in the EPI and ID main tasks, we performed an extensive manual analysis of system outputs, providing an experienced annotator with a random sample of the events predicted by each of the participating systems. To avoid possible bias, we blinded the inputs so that the annotator could not tell which system had created which annotation or whether the annotation had matched gold or not. To further assess the quality and stability of the original gold standard annotation, we additionally included a similarly blinded sample of annotations drawn from the gold standard. Specifically, for both EPI and ID we selected for evaluation a random sample of 100 events from each of the seven final submissions as well as the gold data, for a total of 800 events for each task. These 1,600 events were then blindly evaluated with instructions to mark each as one of *correct*, *acceptable *or *incorrect*. The first of these labels identifies an event as, informally, "as good as gold": a complete and accurate representation of the targeted aspects of the relevant biological claim with respect to the task annotation guidelines. The label *acceptable *specifies that an event is an acceptable representation of the relevant central biological statement, but either incomplete or at variance with the standard guidelines in some secondary aspect. The last label was used for any candidate for which the first two labels were not applicable. As for the system combination, we performed this evaluation for core task subsets as the majority of participants did not attempt the full task.

Table 19 shows the results of the manual analysis for the EPI and ID tasks. We first note that the evaluation supports the estimates of high gold annotation quality: in total, only four of 200 blindly evaluated gold events were marked incorrect. The difference in the number of "accurate" judgments supports a view received also from annotators' informal impressions that the EPI task targets are very strictly defined, while those of the ID task require more interpretation of the guidelines.

**Table 19:** Manual analysis results

	EPI					ID					
	Manual			Gold standard		Manual				Gold standard	
Source	**Corr**.	**Acc**.	**Inc**.	Match	**Prec**.	Source	**Corr**.	**Acc**.	**Inc**.	Match	**Prec**.
GOLD	96	1	3	100	100.0	GOLD	94	5	1	100	100.0

MSR-NLP	87	1	12	83	77.60	FAUST	83	7	10	68	66.35
FAUST	85	0	15	79	80.25	UMass	77	4	19	66	62.39
ConcordU	83	0	17	79	76.71	Stanford	67	7	26	55	56.37
UMass	80	0	20	74	73.30	PNNL	68	4	28	49	52.62
UTurku	75	0	25	72	69.20	UTurku	61	8	31	38	49.91
Stanford	74	0	26	68	70.22	ConcordU	55	5	40	34	43.37
CCP-BTMG	71	0	29	65	63.37	PredX	42	5	53	36	35.18

Number of manually evaluated events judged correct (Corr.), acceptable (Acc.) and incorrect (Inc.) out of samples of 100, with number matching gold annotation (Match) and, for reference, core task precision (Prec.). Analysis performed independently for EPI and ID tasks.

The evaluation shows good correlation with the results of evaluation against gold: a ranking of systems by the number of events judged correct or acceptable produces no instances of disagreement with ranking by the number of matches with gold in the samples. However, despite high correlation, the manual evaluation results indicate systematic differences between the quality of system outputs as perceived in manual evaluation and the results of evaluation against gold. For EPI, the average precision of the systems over the 700 selected events is 74.3% from evaluation against gold while manual evaluation of the same events suggests 79.3% are correct (79.6% correct or acceptable). This effect is consistent also when examining the individual samples of 100 events for each system separately. Accepting (for the moment) the manual evaluation as "ground truth", this result indicates that approximately 20% of events appearing as false positive in evaluation against gold are in fact correct.

For ID, while the average precision for the sample of 700 events is 49.2% in comparison against gold, the manual evaluation suggests that 64.7% of the events are correct. This discrepancy between the two evaluations is larger than that for EPI also in relative terms: with reference to the results of the manual evaluation, 30% of "false positive" events in gold evaluation are correct. Unlike in the EPI evaluation, a nontrivial amount of ID events were marked acceptable; the evaluation suggests that less than 30% of all event predictions are incorrect, a striking contrast to the over 50% estimate from comparison to gold.

Some of these differences are almost certainly due to events missing from gold. However, given the very high precision of gold events measured also in this evaluation, the lack of factors that could make errors of omission up to an order of magnitude more common than errors of commission in gold annotation, and the support from the relative number of "accurate" markings, we find it likely that the observed discrepancy is primarily due to cases that genuinely permit more than one acceptable analysis.

While the trend that this evaluation shows was expected and similar results have been reported previously (e.g. [25]), the specific estimates of the magnitude of the discrepancy between evaluation of event extraction outputs against a fixed gold standard and the perceived quality in manual evaluation is a novel result. This difference was found to be substantial in particular for ID, especially when taking into account cases that are acceptable although not entirely correct. This evaluation indicates that the quality of event extraction results perceived by users may be considerably higher than the primary shared task evaluation would suggest.

## Conclusions

We have presented the preparation, resources, results and analysis of three tasks of the BioNLP Shared Task 2011: the main tasks on Epigenetics and Post-translational modifications (EPI) and Infectious Diseases (ID) and the supporting task on Entity Relations (REL).

The two main tasks were each based on newly annotated corpus resources developed for the purposes of the shared task. For the EPI task, we annotated 1,200 publication abstracts selected as a balanced sample of all PubMed citations regarding the selected event types. For ID, a corpus of 30 full-text publications on the two-component systems subdomain of infectious diseases was created in a collaboration of event annotation and domain experts, adapting and extending the BioNLP'09 Shared Task (ST'09) event representation to the domain. Data for the supporting task REL was created by extending previously introduced GENIA corpus annotations.

The EPI task results demonstrated that the core extraction target of identifying statements of 14 different gene/protein modification types can be reliably addressed by current event extraction methods, with two systems approaching 70% F-score for this task. We further demonstrated that a simple system combination can further improve on this level of performance, reducing error rate by approximately 10%. Challenges remain in detecting statements regarding the catalysis of modification events as well as in resolving the full detail of such events, a task attempted by only one EPI task participant and at which performance remains below 55% F-score.

For the ID task, despite the novel challenges of full papers, four new entity types, extension of event scopes and the introduction of a new event category for high-level processes, the highest results achieved by the seven participating teams were comparable to the state-of-the-art performance on the established ST'09 data, showing that the event extraction approach and present systems generalize well and demonstrating the feasibility of event extraction for the infectious diseases domain. Analysis of the results suggested further opportunities for improving extraction performance, and a system combination experiment demonstrated that even comparatively simple combination approaches can realize further benefits. Finally, manual evalutation of system outputs suggested that many events identified as false positives in evaluation against the gold standard data may nevertheless be acceptable from a user perspective.

Both the EPI and ID main tasks were designed from the perspective of supporting specific biocuration tasks. We believe successful EPI systems have potential to offer further support for existing protein modification database curation as well as for novel efforts targeting specifically epigenetics-related modifications. The resources, tools and methods developed in the ID task are currently being adopted in the development of the Pathosystems Resource Integration Center (PATRIC) [104]. Present and future advances in these tasks can thus assist biologists in efforts of substantial scientific and public health interest.

As was done after the BioNLP'09 shared task, we have made the data, tools and resources for all three task introduced in this paper open to all interested parties to encourage further study of event extraction. The tasks continue as an open shared challenges accessible through the shared task website, http://www.bionlp-st.org/.

## Competing interests

The authors declare that they have no competing interests.

## Authors' contributions

SP and TO conceived of the tasks. SP coordinated the ID and REL task organization, drafted the manuscript, and implemented evaluation and support tools. TO coordinated the EPI task organization and led the EPI, ID and REL annotation efforts. RR, DS, CM and CW contributed to the design of the ID task, RR created the ID task initial automatic annotation, and DS, CM, CW and TO created the ID task manual annotation. BS and SA participated in the design and coordination of the ID task and JT participated in the design and coordination of the overall shared task. All authors read and approved the final manuscript.

## References

[B1] Ananiadou S Kell DB Tsujii J Text mining and its potential applications in systems biology Trends in Biotechnology 2006 24 12 571 579 10.1016/j.tibtech.2006.10.002 17045684

[B2] Zweigenbaum P Demner-Fushman D Yu H Cohen K Frontiers of biomedical text mining: current progress Briefings in Bioinformatics 2007 8 5 358 10.1093/bib/bbm045 17977867 PMC2516302

[B3] Ananiadou S Pyysalo S Tsujii J Kell DB Event extraction for systems biology by text mining the literature Trends in Biotechnology 2010 28 7 381 390 10.1016/j.tibtech.2010.04.005 20570001

[B4] Simpson MS Demner-Fushman D Biomedical Text Mining: A Survey of Recent Progress Mining Text Data 2012 465 517

[B5] Pyysalo S Ginter F Heimonen J Björne J Boberg J Järvinen J Salakoski T BioInfer: A Corpus for Information Extraction in the Biomedical Domain BMC Bioinformatics 2007 8 50 http://w02.biomedcentral.com/1471-2105/8/50 10.1186/1471-2105-8-50 PMC1808065 17291334

[B6] Kim JD Ohta T Tsujii J Corpus annotation for mining biomedical events from literature BMC Bioinformatics 2008 9 10 10.1186/1471-2105-9-10 18182099 PMC2267702

[B7] Thompson P Iqbal S McNaught J Ananiadou S Construction of an annotated corpus to support biomedical information extraction BMC Bioinformatics 2009 10 349 10.1186/1471-2105-10-349 19852798 PMC2774701

[B8] Ohta T Pyysalo S Miwa M Kim JD Tsujii J Event Extraction for Post-Translational Modifications Proceedings of BioNLP'10 2010 19 27 http://aclweb.org/anthology-new/W/W10/W10-1903.pdf

[B9] Pyysalo S Ohta T Miwa M Tsujii J Towards Exhaustive Protein Modification Event Extraction Proceedings of the BioNLP 2011 Workshop 2011 Portland, Oregon: Association for Computational Linguistics http://aclweb.org/anthology-new/W/W11/W11-0215.pdf

[B10] Ohta T Pyysalo S Miwa M Tsujii J Event extraction for DNA methylation Journal of Biomedical Semantics 2011 2 Suppl 5 S2 http://www.jbiomedsem.com/content/2/S5/S2 10.1186/2041-1480-2-S5-S2 22166595 PMC3239302

[B11] Kim JD Ohta T Pyysalo S Kano Y Tsujii J Overview of BioNLP'09 Shared Task on Event Extraction Proceedings of Natural Language Processing in Biomedicine (BioNLP) NAACL 2009 Workshop 2009 1 9 http://aclweb.org/anthology-new/W/W09/W09-1401.pdf

[B12] Chinchor N Overview of MUC-7/MET-2 Message Understanding Conference (MUC-7) Proceedings 1998

[B13] Strassel S Przybocki M Peterson K Song Z Maeda K Linguistic Resources and Evaluation Techniques for Evaluation of Cross-Document Automatic Content Extraction Proceedings of the 6th International Conference on Language Resources and Evaluation (LREC 2008) 2008

[B14] Kim JD, Ohta T, Tsuruoka Y, Tateisi Y, Collier N Introduction to the bio-entity recognition task at JNLPBA Proceedings of COLING 2004 International Joint workshop on Natural Language Processing in Biomedicine and its Applications (NLPBA/BioNLP) 2004 Geneva, Switzerland http://www.aclweb.org/anthology-new/W/W04/W04-1213.pdf

[B15] Nédellec C Cussens J, Nédellec C Learning Language in Logic - Genic Interaction Extraction Challenge Proceedings of the 4th Learning Language in Logic Workshop (LLL05) 2005 31 37

[B16] Hersh W Cohen A Lynn R Roberts P TREC 2007 Genomics track overview Proceeding of the Sixteenth Text REtrieval Conference 2007

[B17] Hirschman L Yeh A Blaschke C Valencia A Overview of BioCreAtIvE: critical assessment of information extraction for biology BMC Bioinformatics 2005 6 Suppl.1 S1 15960821 10.1186/1471-2105-6-S1-S1 PMC1869002

[B18] Krallinger M Morgan A Smith L Leitner F Tanabe L Wilbur J Hirschman L Valencia A Evaluation of text-mining systems for biology: overview of the Second BioCreative community challenge Genome biology 2008 9 Suppl 2 S1 10.1186/gb-2008-9-s2-s1 18834487 PMC2559980

[B19] Arighi C Lu Z Krallinger M Cohen K John Wilbur W Valencia A Hirschman L Wu C Overview of the BioCreative III Workshop BMC Bioinformatics 2011 12 1 9 22151647 10.1186/1471-2105-12-S8-S1 PMC3269932

[B20] Poon H Vanderwende L Joint Inference for Knowledge Extraction from Biomedical Literature Proceedings of NAACL-HLT'10 2010 813 821 http://www.aclweb.org/anthology-new/N/N10/N10-1123.pdf

[B21] Vlachos A Two Strong Baselines for the BioNLP 2009 Event Extraction Task Proceedings of BioNLP'10 2010 1 9 http://aclweb.org/anthology-new/W/W10/W10-1901.pdf

[B22] Miwa M Pyysalo S Hara T Tsujii J A Comparative Study of Syntactic Parsers for Event Extraction Proceedings of BioNLP'10 2010 37 45 http://aclweb.org/anthology-new/W/W10/W10-1905.pdf

[B23] Liu H Blouin C Kešelj V Biological event extraction using subgraph matching Proceedings of the Fourth Symposium on Semantic Mining in Biomedicine SMBM 2010 2010 110 115

[B24] Yoshikawa K Riedel S Hirao T Asahara M Matsumoto Y Coreference based event-argument relation extraction on biomedical text Journal of Biomedical Semantics 2011 2 Suppl 5 S6 http://www.jbiomedsem.com/content/2/S5/S6 10.1186/2041-1480-2-S5-S6 22166257 PMC3239306

[B25] Björne J Ginter F Pyysalo S Tsujii J Salakoski T Complex event extraction at PubMed scale Bioinformatics 2010 26 12 i382 390 10.1093/bioinformatics/btq180 20529932 PMC2881365

[B26] Van Landeghem S Ginter F Van de Peer Y Salakoski T EVEX: A PubMed-Scale Resource for Homology-Based Generalization of Text Mining Predictions Proceedings of BioNLP 2011 Workshop 2011 Portland, Oregon, USA: Association for Computational Linguistics 28 37 http://www.aclweb.org/anthology/W11-0204.pdf

[B27] Ohta T Tateisi Y Mima H Tsujii J GENIA corpus: an annotated research abstract corpus in molecular biology domain Proceedings of the Human Language Technology Conference (HLT 2002), San Diego, California 2002 73 77

[B28] Kim JD Pyysalo S Ohta T Bossy R Tsujii J Overview of BioNLP Shared Task 2011 Proceedings of the BioNLP 2011 Workshop Companion Volume for Shared Task 2011 Portland, Oregon: Association for Computational Linguistics http://aclweb.org/anthology-new/W/W11/W11-1801.pdf

[B29] Tsujii J Kim JD Pyysalo S Proceedings of BioNLP Shared Task 2011 Workshop 2011 Portland, Oregon, USA: Association for Computational Linguistics http://www.aclweb.org/anthology/W11-18.pdf

[B30] Ohta T Pyysalo S Tsujii J Overview of the Epigenetics and Post-translational Modifications (EPI) task of BioNLP Shared Task 2011 Proceedings of the BioNLP 2011 Workshop Companion Volume for Shared Task 2011 Portland, Oregon: Association for Computational Linguistics http://aclweb.org/anthology-new/W/W11/W11-1803.pdf

[B31] Pyysalo S Ohta T Rak R Sullivan D Mao C Wang C Sobral B Tsujii J Ananiadou S Overview of the Infectious Diseases (ID) task of BioNLP Shared Task 2011 Proceedings of BioNLP 2011 2011 http://aclweb.org/anthology-new/W/W11/W11-1804.pdf 10.1186/1471-2105-13-S11-S2 PMC3384257 22759456

[B32] Pyysalo S Ohta T Tsujii J Overview of the Entity Relations (REL) supporting task of BioNLP Shared Task 2011 Proceedings of BioNLP Shared Task 2011 Workshop 2011 Portland, Oregon, USA: Association for Computational Linguistics 83 88 http://www.aclweb.org/anthology/W11-1812.pdf

[B33] Holliday R The inheritance of epigenetic defects Science 1987 238 163 170 10.1126/science.3310230 3310230

[B34] Jaenisch R Bird A Epigenetic regulation of gene expression: how the genome integrates intrinsic and environmental signals Nature Genetics 2003 33 245 254 http://dx.doi.org/10.1038/ng1089 10.1038/ng1089 12610534

[B35] Witze ES Old WM Resing KA Ahn NG Mapping protein post-translational modifications with mass spectrometry Nature Methods 2007 4 798 806 10.1038/nmeth1100 17901869

[B36] Stock J Ninfa A Stock A Protein phosphorylation and regulation of adaptive responses in bacteria Microbiology and Molecular Biology Reviews 1989 53 4 450 http://www.ncbi.nlm.nih.gov/pubmed/2556636 10.1128/mr.53.4.450-490.1989 PMC372749 2556636

[B37] Barford D Das A Egloff M The structure and mechanism of protein phosphatases: insights into catalysis and regulation Annual review of biophysics and biomolecular structure 1998 27 133 164 10.1146/annurev.biophys.27.1.133 9646865

[B38] Glickman M Ciechanover A The ubiquitin-proteasome proteolytic pathway: destruction for the sake of construction Physiological reviews 2002 82 2 373 11917093 10.1152/physrev.00027.2001

[B39] Riggs A X inactivation, differentiation, and DNA methylation Cytogenetic and Genome Research 1975 14 9 25 10.1159/000130315 1093816

[B40] Holliday R Pugh J DNA modification mechanisms and gene activity during development Science 1975 187 226 232 10.1126/science.1111098 1111098

[B41] Hu ZZ Narayanaswamy M Ravikumar KE Vijay-Shanker K Wu CH Literature mining and database annotation of protein phosphorylation using a rule-based system Bioinformatics 2005 21 11 2759 2765 http://bioinformatics.oxfordjournals.org/cgi/content/abstract/21/11/2759 10.1093/bioinformatics/bti390 15814565

[B42] Narayanaswamy M Ravikumar KE Vijay-Shanker K Beyond the clause: extraction of phosphorylation information from medline abstracts Bioinformatics 2005 21 suppl.1 i319 327 http://bioinformatics.oxfordjournals.org/cgi/content/abstract/21/suppl_1/i319 15961474 10.1093/bioinformatics/bti1011

[B43] Yuan X Hu Z Wu H Torii M Narayanaswamy M Ravikumar K Vijay-Shanker K Wu C An online literature mining tool for protein phosphorylation Bioinformatics 2006 22 13 1668 10.1093/bioinformatics/btl159 16644790

[B44] Lee H Yi GS Park JC E3Miner: a text mining tool for ubiquitin-protein ligases Nucl Acids Res 2008 36 suppl.2 W416 422 http://nar.oxfordjournals.org/cgi/content/abstract/36/suppl_2/W416 18483079 10.1093/nar/gkn286 PMC2447767

[B45] Buyko E Faessler E Wermter J Hahn U Event Extraction from Trimmed Dependency Graphs Proceedings of BioNLP Shared Task 2009 2009 19 27 http://aclweb.org/anthology-new/W/W09/W09-1403.pdf

[B46] Thomason P Kay R Eukaryotic signal transduction via histidine-aspartate phosphorelay J Cell Sci 2000 113 18 3141 3150 10954413 10.1242/jcs.113.18.3141

[B47] Mascher T Helmann JD Unden G Stimulus Perception in Bacterial Signal Transducing Histidine Kinases Microbiol Mol Biol Rev 2006 70 4 910 938 10.1128/MMBR.00020-06 17158704 PMC1698512

[B48] Krell T Lacal J Busch A Silva-Jiménez H Guazzaroni ME Ramos JL Bacterial Sensor Kinases: Diversity in the Recognition of Environmental Signals Annual Review of Microbiology 2010 64 539 559 10.1146/annurev.micro.112408.134054 20825354

[B49] Wang C Kemp J Da Fonseca IO Equi RC Sheng X Charles TC Sobral BWS Sinorhizobium meliloti 1021 Loss-of-Function Deletion Mutation in chvI and Its Phenotypic Characteristics Molecular Plant-Microbe Interactions 2010 23 2 153 160 10.1094/MPMI-23-2-0153 20064059

[B50] Gotoh Y Eguchi Y Watanabe T Okamoto S Doi A Utsumi R Two-component signal transduction as potential drug targets in pathogenic bacteria Current Opinion in Microbiology 2010 13 2 232 239 [Cell regulation] 10.1016/j.mib.2010.01.008 20138000

[B51] Kim JD Ohta T Pyysalo S Kano Y Tsujii J Extracting bio-molecular events from literature - the BioNLP'09 shared task Computational Intelligence 2011 27 4 513 540 10.1111/j.1467-8640.2011.00398.x

[B52] Nguyen N Kim JD Tsujii J Overview of the Protein Coreference task in BioNLP Shared Task 2011 Proceedings of the BioNLP 2011 Workshop Companion Volume for Shared Task 2011 Portland, Oregon Association for Computational Linguistics http://aclweb.org/anthology-new/W/W11/W11-1811.pdf

[B53] Kim JD Nguyen N Wang Y Tsujii J Takagi T Yonezawa A The Genia Event (GE) and Protein Coreference (CO) tasks of BioNLP Shared Task 2011 BMC Bioinformatics 2012 13 suppl. 8 S1 10.1186/1471-2105-13-S11-S1 PMC3384256 22759455

[B54] Kim JD Wang Y Takagi T Yonezawa A Overview of the Genia Event task in BioNLP Shared Task 2011 Proceedings of the BioNLP 2011 Workshop Companion Volume for Shared Task 2011 Portland, Oregon: Association for Computational Linguistics http://aclweb.org/anthology-new/W/W11/W11-1802.pdf

[B55] Yeh A Morgan A Colosimo M Hirschman L BioCreAtIvE Task 1A: gene mention finding evaluation BMC Bioinformatics 2005 6 Suppl 1 S2 10.1186/1471-2105-6-S1-S2 15960832 PMC1869012

[B56] Wilbur J Smith L Tanabe L Hirschman L, Krallinger M, Valencia A BioCreative 2. Gene Mention Task Proceedings of Second BioCreative Challenge Evaluation Workshop 2007 7 16

[B57] Morgan A Lu Z Wang X Cohen A Fluck J Ruch P Divoli A Fundel K Leaman R Hakenberg J Overview of BioCreative II gene normalization Genome biology 2008 9 Suppl 2 S3 10.1186/gb-2008-9-s2-s3 18834494 PMC2559987

[B58] Rebholz-Schuhmann D Yepes A Li C Kafkas S Lewin I Kang N Corbett P Milward D Buyko E Beisswanger E Hornbostel K Kouznetsov A Witte R Laurila J Baker C Kuo CJ Clematide S Rinaldi F Farkas R Mora G Hara K Furlong LI Rautschka M Neves M Pascual-Montano A Wei Q Collier N Chowdhury M Lavelli A Berlanga R Morante R Van Asch V Daelemans W Marina J van Mulligen E Kors J Hahn U Assessment of NER solutions against the first and second CALBC Silver Standard Corpus Journal of Biomedical Semantics 2011 2 Suppl 5 S11 http://www.jbiomedsem.com/content/2/S5/S11 10.1186/2041-1480-2-S5-S11 22166494 PMC3239301

[B59] Leaman R Gonzalez G BANNER: an executable survey of advances in biomedical named entity recognition Pacific Symposium on Biocomputing 2008 652 663 http://view.ncbi.nlm.nih.gov/pubmed/18229723 18229723

[B60] Hakenberg J Plake C Leaman R Schroeder M Gonzalez G Inter-species normalization of gene mentions with GNAT Bioinformatics 2008 24 16 i126 10.1093/bioinformatics/btn299 18689813

[B61] Wermter J Tomanek K Hahn U High-performance gene name normalization with GeNo Bioinformatics 2009 25 6 815 10.1093/bioinformatics/btp071 19188193

[B62] Wei CH Kao HY Cross-species gene normalization by species inference BMC bioinformatics 2011 12 Suppl 8 S5 10.1186/1471-2105-12-S8-S5 22151999 PMC3269940

[B63] Hoehndorf R Ngonga Ngomo AC Pyysalo S Ohta T Oellrich A Rebholz-Schuhmann D Ontology design patterns to disambiguate relations between genes and gene products in GENIA Journal of Biomedical Semantics 2011 2 Suppl 5 S1 http://www.jbiomedsem.com/content/2/S5/S1 10.1186/2041-1480-2-S5-S1 22166341 PMC3239299

[B64] Vincze V Szarvas G Farkas R Móra G Csirik J The BioScope corpus: biomedical texts annotated for uncertainty, negation and their scopes BMC bioinformatics 2008 9 Suppl 11 S9 10.1186/1471-2105-9-S11-S9 19025695 PMC2586758

[B65] Farkas R Vincze V Móra G Csirik J Szarvas G The CoNLL-2010 shared task: learning to detect hedges and their scope in natural language text Proceedings of the Fourteenth Conference on Computational Natural Language Learning--Shared Task 2010 Association for Computational Linguistics 1 12 http://aclweb.org/anthology-new/W/W10/W10-3001.pdf

[B66] Ashburner M Ball C Blake J Botstein D Butler H Cherry J Davis A Dolinski K Dwight S Eppig J Harris M Hill D Issel-Tarver L Kasarskis A Lewis S Matese J Richardson J Ringwald M Rubin G Sherlock G Gene ontology: tool for the unification of biology Nature genetics 2000 25 25 29 10.1038/75556 10802651 PMC3037419

[B67] Pyysalo S Ohta T Kim JD Tsujii J Static Relations: a Piece in the Biomedical Information Extraction Puzzle Proceedings of Natural Language Processing in Biomedicine (BioNLP) NAACL 2009 Workshop 2009 Boulder, Colorado: Association for Computational Linguistics 1 9 http://aclweb.org/anthology-new/W/W09/W09-1301.pdf

[B68] Ohta T Pyysalo S Kim JD Tsujii J A re-evaluation of biomedical named entity-term relations Journal of Bioinformatics and Computational Biology (JBCB) 2010 8 5 917 928 10.1142/S0219720010005014 20981895

[B69] Van Landeghem S Pyysalo S Ohta T Van de Peer Y Integration of Static Relations to Enhance Event Extraction from Text Proceedings of the 2010 Workshop on Biomedical Natural Language Processing 2010 Uppsala, Sweden: Association for Computational Linguistics 144 152 http://www.aclweb.org/anthology/W10-1921.pdf

[B70] Winston ME Chaffin R Herrmann D A taxonomy of part-whole relations Cognitive Science 1987 11 4 417 444 10.1207/s15516709cog1104_2

[B71] Protein Information Resource (PIR) http://pir.georgetown.edu

[B72] Wu CH Yeh LSL Huang H Arminski L Castro-Alvear J Chen Y Hu Z Kourtesis P Ledley RS Suzek BE Vinayaka C Zhang J Barker WC The Protein Information Resource Nucl Acids Res 2003 31 345 347 10.1093/nar/gkg040 12520019 PMC165487

[B73] PubMeth: Reviewed methylation database in cancer http://www.pubmeth.org/

[B74] Ongenaert M Van Neste L De Meyer T Menschaert G Bekaert S Van Criekinge W PubMeth: a cancer methylation database combining text-mining and expert annotation Nucl Acids Res 2008 36 suppl 1 D842 846 17932060 10.1093/nar/gkm788 PMC2238841

[B75] Tanabe L Xie N Thom L Matten W Wilbur J GENETAG: a tagged corpus for gene/protein named entity recognition BMC Bioinformatics 2005 6 Suppl 1 S3 10.1186/1471-2105-6-S1-S3 15960837 PMC1869017

[B76] Wang Y Kim JD Sætre R Pyysalo S Tsujii J Investigating heterogeneous protein annotations toward cross-corpora utilization BMC Bioinformatics 2009 10 403 http://w02.biomedcentral.com/1471-2105/10/403 [ISSN: 1471-2105] 10.1186/1471-2105-10-403 PMC2804683 19995463

[B77] Ohta T Kim JD Pyysalo S Wang Y Tsujii J Incorporating GENETAG-style annotation to GENIA corpus Proceedings of BioNLP'09 2009 106 107 http://aclweb.org/anthology-new/W/W09/W09-1313.pdf

[B78] Ohta T Kim JD Tsujii J Guidelines for event annotation 2007 Tech rep, Tsujii Laboratory, University of Tokyo

[B79] Pyysalo S Ohta T Cho HC Sullivan D Mao C Sobral B Tsujii J Ananiadou S Towards Event Extraction from Full Texts on Infectious Diseases Proceedings of BioNLP'10 2010 132 140 http://aclweb.org/anthology-new/W/W10/W10-1919.pdf

[B80] Ananiadou S Sullivan D Black W Levow GA Gillespie JJ Mao C Pyysalo S Kolluru B Tsujii J Sobral B Named Entity Recognition for Bacterial Type IV Secretion Systems PLoS ONE 2011 6 3 e14780 10.1371/journal.pone.0014780 21468321 PMC3066171

[B81] GENIA Sentence Splitter https://github.com/TsujiiLaboratory/geniass

[B82] Sasaki Y Tsuruoka Y McNaught J Ananiadou S How to make the most of NE dictionaries in statistical NER BMC bioinformatics 2008 9 Suppl 11 http://w01.biomedcentral.com/1471-2105/9/S11/S5/10.1186/1471-2105-9-S11-S5 PMC2586754 19025691

[B83] Gerner M Nenadic G Bergman CM LINNAEUS: a species name identification system for biomedical literature BMC bioinformatics 2010 11 85+ 10.1186/1471-2105-11-85 20149233 PMC2836304

[B84] Corbett P Murray-Rust P High-Throughput Identification of Chemistry in Life Science Texts Computational Life Sciences II 2006 107 118

[B85] Pyysalo S Ohta T Rak R Sullivan D Mao C Wang C Sobral B Tsujii J Ananiadou S Annotation Guidelines for Infectious Diseases Event Corpus Tech rep, Tsujii Laboratory, University of Tokyo 2011

[B86] Tateisi Y Yakushiji A Ohta T Tsujii J Syntax Annotation for the GENIA corpus Proceedings of IJCNLP'05 2005 222 227

[B87] Noreen EW Computer-Intensive Methods for Testing Hypotheses: An Introduction Wiley 1989

[B88] Chinchor N The statistical significance of the MUC-4 results Proceedings of the Fourth Message Understanding Conference (MUC-4) 1992 Association for Computational Linguistics 30 50

[B89] Riedel S McClosky D Surdeanu M McCallum A Manning C Model Combination for Event Extraction in BioNLP 2011 Proceedings of the BioNLP 2011 Workshop Companion Volume for Shared Task 2011 Portland, Oregon: Association for Computational Linguistics

[B90] McClosky D Surdeanu M Manning C Event Extraction as Dependency Parsing for BioNLP 2011 Proceedings of the BioNLP 2011 Workshop Companion Volume for Shared Task 2011 Portland, Oregon: Association for Computational Linguistics http://aclweb.org/anthology-new/W/W11/W11-1808.pdf

[B91] Riedel S McCallum A Robust Biomedical Event Extraction with Dual Decomposition and Minimal Domain Adaptation Proceedings of the BioNLP 2011 Workshop Companion Volume for Shared Task 2011 Portland, Oregon: Association for Computational Linguistics http://aclweb.org/anthology-new/W/W11/W11-1806.pdf

[B92] Björne J Salakoski T Generalizing Biomedical Event Extraction Proceedings of the BioNLP 2011 Workshop Companion Volume for Shared Task 2011 Portland, Oregon: Association for Computational Linguistics http://aclweb.org/anthology-new/W/W11/W11-1807.pdf

[B93] Quirk C Choudhury P Gamon M Vanderwende L MSR-NLP Entry in BioNLP Shared Task 2011 Proceedings of the BioNLP 2011 Workshop Companion Volume for Shared Task 2011 Portland, Oregon: Association for Computational Linguistics http://aclweb.org/anthology-new/W/W11/W11-1828.pdf

[B94] McGrath L Domico K Corley C Webb-Robertson BJ Complex Biological Event Extraction from Full Text using Signatures of Linguistic and Semantic Features Proceedings of the BioNLP 2011 Workshop Companion Volume for Shared Task 2011 Portland, Oregon: Association for Computational Linguistics http://aclweb.org/anthology-new/W/W11/W11-1825.pdf

[B95] Charniak E Johnson M Coarse-to-Fine n-Best Parsing and MaxEnt Discriminative Reranking Proceedings of the 43rd Annual Meeting of the Association for Computational Linguistics (ACL'05) 2005 173 180 http://aclweb.org/anthology-new/W/W11/W11-1818.pdf

[B96] McClosky D Any Domain Parsing: Automatic Domain Adaptation for Natural Language Parsing PhD thesis 2009 Department of Computer Science, Brown University http://www.aclweb.org/anthology-new/P/P05/P05-1022.pdf

[B97] de Marneffe MC MacCartney B Manning CD Generating Typed Dependency Parses from Phrase Structure Parses Proceedings of the Fifth International Conference on Language Resources and Evaluation (LREC'06) 2006 449 454

[B98] Stenetorp P Topić G Pyysalo S Ohta T Kim JD Tsujii J BioNLP Shared Task 2011: Supporting Resources Proceedings of the BioNLP 2011 Workshop Companion Volume for Shared Task 2011 Portland, Oregon: Association for Computational Linguistics

[B99] Kilicoglu H Bergler S Adapting a General Semantic Interpretation Approach to Biological Event Extraction Proceedings of the BioNLP 2011 Workshop Companion Volume for Shared Task 2011 Portland, Oregon: Association for Computational Linguistics http://aclweb.org/anthology-new/W/W11/W11-1816.pdf

[B100] Van Landeghem S Abeel T De Baets B Van de Peer Y Detecting Entity Relations as a Supporting Task for Bio-Molecular Event Extraction Proceedings of BioNLP Shared Task 2011 Workshop 2011 Portland, Oregon, USA: Association for Computational Linguistics 147 148 http://www.aclweb.org/anthology/W11-1821.pdf

[B101] Le Minh Q Nguyen Truong S Ho Bao Q A pattern approach for Biomedical Event Annotation Proceedings of the BioNLP 2011 Workshop Companion Volume for Shared Task 2011 Portland, Oregon: Association for Computational Linguistics

[B102] Tikk D Thomas P Palaga P Hakenberg J Leser U A Comprehensive Benchmark of Kernel Methods to Extract Protein--Protein Interactions from Literature PLoS Comput Biol 2010 6 7 e1000837 10.1371/journal.pcbi.1000837 20617200 PMC2895635

[B103] Miwa M Pyysalo S Hara T Tsujii J Evaluating dependency representation for event extraction Proceedings of COLING'10 2010 779 787

[B104] Pathosystems Resource Integration Center (PATRIC) http://patricbrc.org 10.1093/nar/gkl858 PMC1669763 17142235

[B105] Stenetorp P Pyysalo S Topić G Ohta T Ananiadou S Tsujii J brat: a Web-based Tool for NLP-Assisted Text Annotation Proceedings of the Demonstrations Session at EACL 2012 2012

[B106] brat rapid annotation tool http://brat.nlplab.org

[B107] McClosky D Riedel S Surdeanu M Manning C McCallum A Combining joint models for biomedical event extraction BMC Bioinformatics 2012 13 suppl. 8 S9 10.1186/1471-2105-13-S11-S9 PMC3395172 22759463

[B108] Björne J Heimonen J Ginter F Airola A Pahikkala T Salakoski T Extracting Complex Biological Events with Rich Graph-Based Feature Sets Proceedings of the BioNLP 2009 Workshop Companion Volume for Shared Task 2009 Boulder, Colorado: Association for Computational Linguistics 10 18 http://www.aclweb.org/anthology/W09-1402.pdf

[B109] Björne J Ginter F Salakoski T Generalizing Biomedical Event Extraction BMC Bioinformatics 2012 13 suppl. 8 S4 10.1186/1471-2105-16-S16-S4 PMC4642046 26551925

[B110] Van Landeghem S Björne J Abeel T De Baets B Salakoski T Van de Peer Y Semantically linking molecular entities in literature through entity relationships BMC Bioinformatics 2012 13 suppl 11 S6 10.1186/1471-2105-13-S11-S6 PMC3384255 22759460

[B111] Riedel S Chun HW Takagi T Tsujii J A Markov Logic Approach to Bio-Molecular Event Extraction Proceedings of the BioNLP 2009 Workshop Companion Volume for Shared Task 2009 Boulder, Colorado Association for Computational Linguistics 41 49 http://www.aclweb.org/anthology/W09-1406.pdf

[B112] Riedel S McCallum A Fast and Robust Joint Models for Biomedical Event Extraction Proceedings of the 2011 Conference on Empirical Methods in Natural Language Processing 2011 1 12

[B113] McClosky D Surdeanu M Manning C Event Extraction as Dependency Parsing Proceedings of the 49th Annual Meeting of the Association for Computational Linguistics: Human Language Technologies 2011 Portland, Oregon, USA: Association for Computational Linguistics 1626 1635 http://aclweb.org/anthology-new/D/D11/D11-1001.pdf

[B114] Kilicoglu H Bergler S Syntactic Dependency Based Heuristics for Biological Event Extraction Proceedings of the BioNLP 2009 Workshop Companion Volume for Shared Task 2009 Boulder, Colorado: Association for Computational Linguistics 119 127 http://www.aclweb.org/anthology/W/W09/W09-1418.pdf

[B115] Kilicoglu H Bergler S Biological Event Composition BMC Bioinformatics 2012 13 suppl. 8 S7 10.1186/1471-2105-13-S11-S7 PMC3384260 22759461

[B116] Liu H Komandur R Verspoor K From graphs to events: A subgraph matching approach for information extraction from biomedical text Proceedings of the BioNLP 2011 Workshop Companion Volume for Shared Task 2011 Portland, Oregon: Association for Computational Linguistics

[B117] Stanford CoreNLP - A Suite of Core NLP Tools 2011 http://nlp.stanford.edu/software/corenlp.shtml

[B118] Porter M An algorithm for suffix stripping Program 1980 14 3 130 137 10.1108/eb046814

[B119] Porter M Boulton R Snowball stemmer 2001 http://snowball.tartarus.org

[B120] Sleator DD Temperley D Parsing English with a Link Grammar Tech. Rep. CMU-CS-91-196 1991 Carnegie Mellon University

[B121] Bodenreider O The Unified Medical Language System (UMLS): integrating biomedical terminology Nucleic Acids Research 2004 32 D267 D270 10.1093/nar/gkh061 14681409 PMC308795

